# Temporal representation impairment in developmental dyslexia for unisensory and multisensory stimuli

**DOI:** 10.1111/desc.12977

**Published:** 2020-06-14

**Authors:** Monica Gori, Kinga M. Ober, Francesca Tinelli, Olivier A. Coubard

**Affiliations:** ^1^ U‐VIP Unit for Visually Impaired People Istituto Italiano di Tecnologia Genoa Italy; ^2^ Faculty of Educational Studies Adam Mickiewicz University Poznan Poland; ^3^ Department of Developmental Neuroscience Stella Maris Scientific Institute Pisa Italy; ^4^ The Neuropsychological Laboratory CNS‐Fed Paris France

**Keywords:** Multisensory, Development, integration, Bayesian, Audio, Visual

## Abstract

Dyslexia has been associated with a problem in visual–audio integration mechanisms. Here, we investigate for the first time the contribution of unisensory cues on multisensory audio and visual integration in 32 dyslexic children by modelling results using the Bayesian approach. Non‐linguistic stimuli were used. Children performed a temporal task: they had to report whether the middle of three stimuli was closer in time to the first one or to the last one presented. Children with dyslexia, compared with typical children, exhibited poorer unimodal thresholds, requiring greater temporal distance between items for correct judgements, while multisensory thresholds were well predicted by the Bayesian model. This result suggests that the multisensory deficit in dyslexia is due to impaired audio and visual inputs rather than impaired multisensory processing per se. We also observed that poorer temporal skills correlated with lower reading skills in dyslexic children, suggesting that this temporal capability can be linked to reading abilities.

## Graphical abstract

Multisensory audio visual processing in dyslexia is due to impaired audio and visual inputs rather than impaired multisensory processing per se.

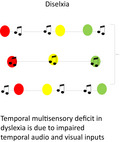

**Highlights**

- We measured audio and visual temporal thresholds in children with dyslexia.
- Auditory, visual and multisensory thresholds were impaired in dyslexic children.
- Multisensory integration was well predicted by the Bayesian model in dyslexic children.
- Children's age and reading abilities correlated with auditory temporal thresholds in dyslexic children.
- Multisensory impairment in dyslexic children is due to a deficit in unisensory audio and visual signals.

## 1 INTRODUCTION

Our five senses provide complementary information about the environment. These combine to yield a single multisensory perception of the world. Reading is an example of multisensory processing. Written words, decoded from vision are associated with sounds decoded from audition during loud reading. Reading aloud is indeed normal at the early stage of learning to read. Later, children, between grades 1–2 and 5–6, learn to read silently. This basic process is impaired in dyslexic people (about 5% of the global population). Considerable research efforts have been directed to understand the processing deficits that lead to dyslexia (Snowling, Bishop, Bishop, & Stothard, 2000; Ramus, 2001; Stein, 2001; Vellutino, Fletcher, Fletcher, Snowling, & Scanlon, 2004; Schulte‐Korne & Bruder, 2010; Leppanen et al., 2012; Hamalainen, Salminen, Salminen, & Leppanen, 2013). Numerous theories have been proposed (Vellutino et al., 2004; Heim et al., 2008) including deficits related to the learning process (Gascon & Goodglass, 1970; Manis et al., 1987), attention problems (Pelham & Ross, 1977; Hari & Renvall, 2001; Shaywitz et al., 2002), phonological deficits (Wagner, 1986; Snowling et al., 2000; Ramus, 2001, 2003; Ramus & Szenkovits, 2008; Kovelman et al., 2012; Ramus, Marshall, Marshall, Rosen, & van der Lely, 2013), magnocellular deficits (Lovegrove, Heddle, Heddle, & Slaghuis, 1980; Galaburda, 1994; Stein & Walsh, 1997; Stein, 2001) and cerebellar deficits (Nicolson & Fawcett, 1990; Nicolson, Fawcett, Fawcett, & Dean, 2001). Different subtypes of dyslexia have been also reported (Heim et al., 2008) suggesting that this reading impairment may be associated with different cognitive problems.

Since reading is a multisensory process, audio and visual multisensory associations have been widely investigated in dyslexic populations. The majority of studies have involved speech sound–grapheme combinations or audio and visual speech stimuli (e.g. Froyen, Van Atteveldt, Van Atteveldt, Bonte, & Blomert, 2008; Froyen, Bonte, Bonte, van Atteveldt, & Blomert, 2009; Froyen, Willems, Willems, & Blomert, 2011; Mittag, Thesleff, Thesleff, Laasonen, & Kujala, 2013). Sensory studies in dyslexia highlighted the difficulty of dyslexic children in perceptual anchoring (i.e. in dynamically constructing stimulus‐specific prediction; Ahissar, 2007; Daikhin, Raviv, Raviv, & Ahissar, 2017). Similarly, deficits were observed in implicit learning and in identifying stimuli when masked by external visual noise (Sperling, Lu, Lu, & Manis, 2004).

Most of the past studies performed to date in dyslexia have investigated separately unisensory or multisensory processing without considering them together. A crucial component for reading is the temporal component of multisensory integration: audio and visual signals need to be synchronized and integrated. Past studies have investigated the integration between audio and visual temporal signals in typical individuals determining the temporal factors that are necessary to integrate audio and visual modalities. These works quantified the temporal binding window, namely the epoch of time within which audio and visual stimuli are likely to be integrated and perceptually bound in typical individuals and showed that this is altered in a series of neurodevelopmental disorders (Wallace & Stevenson, 2014 for a review). Another important component of multisensory integration is the benefit that is provided by the presence of multiple sensory signals. Many recent studies show that our brain is able to integrate unisensory signals in a statistically optimal Bayesian fashion (Ernst & Banks, 2002; Alais & Burr, 2004; Landy, Banks, & Knill, 2011). The Bayesian approach predicts that different sensory inputs are combined after weighting unisensory visual and auditory signals for reliability and predicts an improvement of precision in the multisensory estimation compared with the precision obtained for unisensory estimations. This model has been shown to be a powerful method in predicting multisensory integration in many tasks (Ernst & Banks, 2002; Alais & Burr, 2004; Landy et al., 2011). In children, reliability‐based multisensory integration seems to develop late, after 8–10 years of age; before then, one sense (such as audio or haptic) dominates the other (such as vision) (Gori, Del Viva, Del Viva, Sandini, & Burr, 2008; Gori, Giuliana, Giuliana, Sandini, & Burr, 2012; Gori, Sandini, Sandini, & Burr, 2012). This sensory dominance can be associated with a cross‐sensory calibration that may occur during development for specific sensory modalities over others (Sciutti et al., 2010; Gori, Sciutti, Sciutti, Burr, & Sandini, 2011; Burr & Gori, 2012; Gori, 2015). To date, no studies have quantified the level of audio–visual multisensory integration in dyslexic children while considering the contribution of each unisensory signal on the processing of multisensory temporal signals. To investigate this, we measured in each child unisensory audio and visual temporal perception and their multisensory integration. To quantify the level of multisensory integration, we modelled results using a Bayesian modelling approach. Understanding how unisensory signals are weighted in the multisensory framework of Bayesian integration in dyslexic children can provide important inputs to understanding how (a) visual and audio information is integrated and processed in dyslexic children and (b) how their unisensory and multisensory skills are linked with their reading capabilities.

A non‐linguistic audio and visual temporal bisection task was performed in 32 typical and 32 dyslexic children. Typical children perform this simple temporal task in an adult‐like manner by around 10 years of age (Gori et al., 2012). During this task, the participant has to encode the temporal sequence of three stimuli and compare their temporal relation. Temporal unisensory audio and visual and multisensory conflictual and non‐conflictual conditions were tested. Results were modelled using the Bayesian approach. Comparisons with reading skills and age of children were performed. Results suggest that children with dyslexia, compared to typical children, exhibited poorer unimodal thresholds requiring greater temporal distance between items for correct judgements. Multisensory thresholds were also higher than in typical children but interestingly they were well predicted by the Bayesian model. This result suggests that the multisensory deficit in dyslexia may be associated with an impaired unisensory audio and visual processing rather than a multisensory integration deficit per se. Poorer audio temporal skills were also correlated with lower reading skills in dyslexic children, suggesting a possible link between this temporal capability and reading abilities.

## 2 METHODS

### 2.1 Participants

**FIGURE 1:**
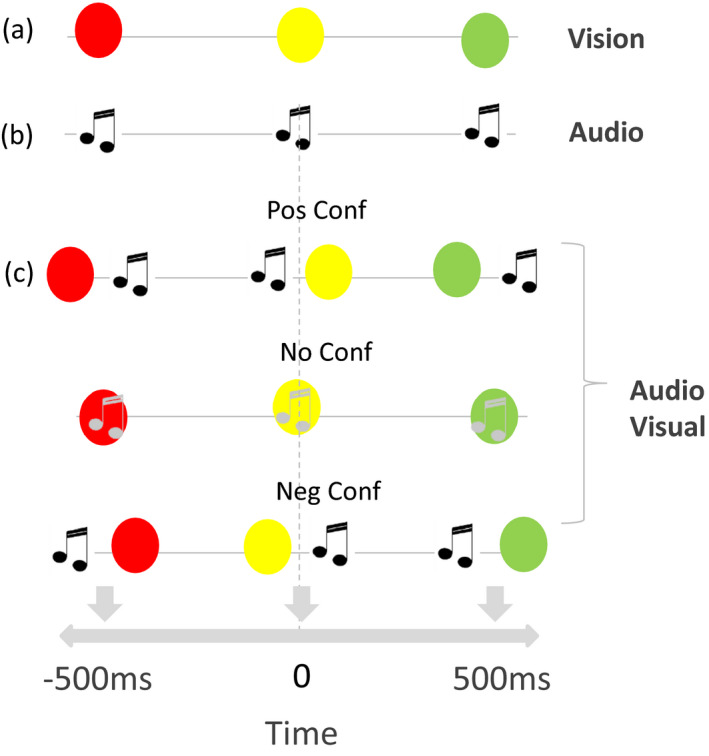
Stimuli. (a) Representation of the visual stimulus. The participant was presented with a sequence of three lights: the first red, the second yellow and the third green. The participant's task was to determine whether the second light appeared closer in time to the first or the last one. (b) Representation of the auditory stimulus. The participant was presented with three sounds. The participant's task was to say if the second sound was presented closer in time to the first or the third one. (c) Representation of the bimodal stimulus. The participant was presented with a sequence of three lights (as in (a)) together with three sounds (like (b)). The visual and the auditory stimuli were presented with a temporal offset of +Δ (=0, ±50 ms) in the second stimulus, −Δ in the first and third (see methods section for more details)

In all, 32 dyslexic children and 32 age‐ and gender‐matched typical children (aged between 7 and 14 years; see Table 1 for more details) performed a visual, audio and bimodal temporal bisection task, illustrated in Figure 1.

**TABLE 1:** Clinical details of subjects

CODE	GENDER	Age in months	Years of education (Lezak, Howieson, Howieson, Bigler, & Tranel, 2012)	Intelligence’ Raven IQ	READING Decoding ^2–3^	READING comprehension ^2–3^
DY01	1	84.53	2	92	358	302
DY02	1	84.59	2	82	432	420
DY03	1	87.49	3	81	332	292
DY04	1	98.69	3	97	378	345
DY05	1	101.66	3	88	312	301
DY06	1	106.49	4	82	310	258
DY07	1	108.56	3	75	373	331
DY08	1	114.66	5	74	280	215
DY09	1	123.39	5	92	244	217
DY10	1	149.39	7	79	202	175
DY11	1	150.36	7	84	182	163
DY12	2	85.43	2	79	330	295
DY13	2	95.10	4	94	292	234
DY14	2	97.92	2	73	362	311
DY15	2	101.69	3	77	325	253
DY16	2	110.69	4	74	298	298
DY17	2	112.33	5	86	250	205
DY18	2	120.66	5	67	275	242
DY19	2	126.62	6	86	192	157
DY20	2	132.49	6	72	195	180
DY21	2	133.46	6	95	228	152
DY22	2	136.62	6	83	235	182
DY23	2	143.43	7	64	213	177
DY24	2	145.95	7	71	214	165
DY25	2	147.33	7	72	257	212
DY26	2	153.16	7	80	201	175
DY27	2	105.46	9	100	Not available	Not available
DY28	2	159.66	5	100	Not available	Not available
DY29	1	123.13	9	96	Not available	Not available
DY30	1	158.76	8	100	Not available	Not available
DY31	1	150.26	8	Not available	Not available	Not available
DY32	1	157.66	7	93	Not available	Not available

Raven IQ	Percentile
Reading decoding	Prolexia decoding in seconds (Polish test)
Reading comprehension	Prolexia comprehension in seconds (Polish test)
Gender	1 = Boy; 2 = Girl
Age in months	Numerical age in months

Children with dyslexia were recruited at the IRCS Fondazione Stella Maris Scientific Institute in Pisa for Italian children and at the Psychological Educational Assessment Centre in Poznan for Polish children. Dyslexic children were tested at the IRCS Fondazione Stella Maris Scientific Institute in Pisa, and at primary schools number 58 Jerzy Kukuczka and at school number 70 Nicolaus Copernicus in Poznań, Poland. Dyslexic children were followed by these structures in terms of assessment and evaluation. All dyslexic children had a history of reading difficulties. All dyslexic children went through medical and psychological examinations before inclusion in the study. Medical examinations included ophthalmologic, otorhinolaryngologic and neurological assessments to exclude aetiology other than dyslexia. The psychological examination was carried out at the IRCCS Fondazione Stella Maris in Calambrone for Italian children and at primary schools number 58 Jerzy Kukuczka and at school number 70 Nicolaus Copernicus in Poznań, Poland. A multidisciplinary team diagnosed dyslexia according to standard tests. Specifically, Polish children underwent the Wechsler Intelligence Scale for Children IV – WISC‐IV (Wechsler, 2014), and a standardized Polish battery to diagnose dyslexia (Bogdanowicz et al., 2008). All Italian dyslexic children underwent the WISC‐IV (Wechsler, 2014), and a standardized Italian battery to diagnose dyslexia (Sartori, Job, Job, & Tressoldi, 2007).

Typical children were recruited from elementary and intermediate schools in Genoa. In typical children, the reading and IQ skills were not collected because of logistic and organizational constraints but both teachers and parents did not report any reading or cognitive impairments in the typical children included in this study. Typical and dyslexic children were matched by age. The average age of dyslexic group was 10 years ± 4 months and gender was balanced (16 girls and 16 boys). Typical children were age and gender matched (average age: 10 years ± 4 months). The age of the two groups was not statistically different (two‐tailed *t* test, *t*(62) = −0.47, *p* < .64, *d* = 0.07).

### 2.2 Reading tests and raven matrices

All dyslexic children showed deficits in reading, despite normal intelligence measured with both Raven Matrices (Raven, Raven, Raven, & Court, 1998) and WISC‐IV (Wechsler, 2014) batteries. Raven Matrices results for IQs are reported in Table 1. All children except two children had an IQ > 70, the remaining two children had IQs = 67 and 64. The performances in our tasks for these two children were comparable to those of the other dyslexic children, suggesting that lower IQ was not associated with poorer temporal skills in our task. In general, no correlation between temporal skills and IQ was observed (see the results section for more details on the correlation analysis performed).

A reading test was also performed in Polish children: the Prolexia Polish test for reading (Ober, Jaskowska, Jaskowska, Jaskowski, & Ober, 1998). Prolexia examines two aspects of reading skills, decoding (44 chain words) and comprehension (31 chain sentences). The results (Table 1) indicate the time in seconds to perform the task: the lower the execution time the better the performance for both tasks (Ober et al., 1998; Brzezinska, 2003).

### 2.3 Ethics

The study was approved by the ethics committees of the local health service in Italy and in Poland. In Italy, the study was approved by the ethics committees of the local health services: Comitato Etico, ASL3 Genovese, Italy, and Comitato Etico, IRCCS Fondazione Stella Maris 36/2010, Pisa, Italy. The study was also approved by the ethics committees of Adam Mickiewicz University (Komisja Etyczna UAM ds. badan naukowych) in agreement with scientific research regulation (directive 2001/20/WE 120 from 4th April, 2001 issued by European Commission). The study was conducted in line with the Declaration of Helsinki.

Parents of participants gave their written consent for the children to participate in the study. The goal of the study, the type of experiment and the possibility to withdraw at any time during the study were explained to the children. In all, 26 of the dyslexic children tested were Polish (DY01 to DY26 in Table 1) and six children were Italian (DY27 to DY32 in Table 1). The study was conducted in line with the Declaration of Helsinki.

### 2.4 Stimuli and procedure

Three different classes of stimuli (visual, auditory and bimodal = both visual and auditory) were presented sequentially for a total duration of 1 s. Participants were required to indicate, by button press, whether the middle stimulus appeared closer in time to the first or the third stimulus (temporal bisection). In the visual task (Figure 1a), the participant was presented with a sequence of three lights: red, yellow and green. The subject had to determine whether the second (yellow) light appeared closer in time to the first (red) or to the last (green) light. Similarly, in the auditory task (Figure 1b), the participant had to indicate whether the second sound was presented closer in time to the first or to the third. In the bimodal task (Figure 1c), the participant was presented with a sequence of three lights associated with three sounds (like the unisensory stimuli). The visual and the auditory stimuli were presented either at the same time or ‘in conflict’, with the auditory stimulus preceding or trailing the visual one. The creation of conflicts was key for making specific predictions and quantifying the weight given to each sensory modality in the multisensory integration process when conflicts between senses were present. The model predicts that the final multisensory estimate is weighted considering the reliability of unisensory conditions and its variance is lowest than that one of the unisensory cues. For example, if we consider that the audio modality is more reliable than the visual one, then the prediction is that in the conflictual condition, the multisensory stimulus should be shifted accordingly with the temporal position of the auditory stimulus which should be perceived in the middle between the first and third stimuli. In contrast, if the visual modality is more reliable, the visual cue will be predicted to be perceived in the middle between the first and the third stimuli. Finally, an ideal observer with exactly equal reliability of visual and auditory judgements of time should perceive the second audio and visual event exactly in‐between the first and second because the conflicting auditory and visual cues should be weighted equally, so their equal offsets in time should average to a zero offset. In all the conditions, the prediction on precision is that the multisensory variance is smaller than the unisensory variances with a higher benefit when both audio and visual variances are equal. The procedure used in this manuscript was identical to that of Gori et al. (Gori et al., 2012). In all bimodal trials, lights were always presented in the same sequence as in the visual task, but the timing of lights and sounds were presented randomly in one of three conditions (Figure 1): (a) the light preceded the sound of 50 ms in the first and third stimuli and the sound preceded the light of 50 ms in the second stimulus; (b) light and sounds were presented simultaneously for all the three stimuli and (c) the sound preceded the light of 50 ms for the first and third stimuli and the light preceded the sound of 50 ms in the second stimulus. Thus, the visual and the auditory stimuli could be presented in conflict or not (Δ = −100; 0; 100 ms): the 100 ms of conflict was obtained by inserting a conflict in the second stimulus of Δ ms (Δ = −50; 0; 50 ms) and an opposite sign of conflict in the first and in the third stimuli (−Δ ms). To estimate the psychometric function, the probability of perceiving the second sound closer to the first one or the third one was calculated by varying the timing of the second stimulus (tone and flash) to span the interval between the first and third stimuli. We used a child‐friendly setup. The visual stimuli were 1° diameter LEDs displayed for 75 ms. Auditory stimuli were 750 Hz tones played for 75 ms. This temporal bisection stimulus was previously used to study temporal representation in typical children and in children with auditory deficits. Using this task, we have highlighted an audio dominance in temporal representation in typical children and adults (Gori et al., 2012) and a deficit in deaf children (Gori, Chilosi, Chilosi, Forli, & Burr, 2017).

To check for the delay in the stimulus presentation, we used a light sensor and microphone to record the delay to compare timing of stimulus presentations as estimated from the Matlab program and the effective sound and/or light presentation estimated through the light sensor and microphone. The delay was estimated for each condition and synchronization between the signals was regulated accordingly. Before data collection, participants were familiarized with the task during two training sessions of 10 trials each (one visual and one audio), where participants indicated, after each presentation of the three stimuli, whether the second appeared earlier or later than the midpoint between the first and third stimuli (as in the main experiment). We provided feedback during these training sessions so participants could learn how to complete the task and minimize errors in their responses. No feedback was given subsequent to training sessions. During the experimental sessions, five different conditions were presented in the following order: vision only, auditory only and the three two‐cue conditions. The total session included 150 trials (30 for each condition). Accurate timing of the visual and auditory stimuli was ensured by setting priority in the operating system to maximum during stimulus presentation to avoid interruptions by other processes.

The time of presentation of the second stimulus (the comparison) was varied using independent quick estimate by sequential testing (QUEST) routines that are a maximum likelihood method (Watson & Pelli, 1983). The stimulus intensity presented at each trial was determined by a statistical estimation of the subject threshold based on all responses from the beginning of the session. After each trial, threshold and point of subjective equality (PSE) were re‐estimated and the stimulus intensity adjusted accordingly. QUEST start from prior information, to construct a probability distribution function (PDF) based on the PDF stimulus intensity most likely to be the subject's threshold. The QUEST estimate was perturbed by adding a random number to ensure that the psychometric function was well sampled over its entire range, important when estimating both the PSE and slope. It also gave participants a few encouraging ‘easy’ trials from time to time (around 20%). In the easy trials, the second stimulus was close to the first or to the third stimulus (less than 100 ms) and the performance of children was predicted to be higher than 80% under this condition. The prior information used in the QUEST, the level of perturbation and the amount of easy trials were estimated using the results obtained in preliminary testing and in our previous experiments performed using the same protocol in typical (Gori et al., 2012) and atypical (Gori et al., 2017) children.

The proportion of trials where the second stimulus was judged closer to the third stimulus was computed for each comparison position in time. Data for each condition were fit using a cumulative Gaussian function, providing the point of subjective equality (PSE) as the time offset for which the second stimulus, on average, appeared to bisect the first and third stimuli and the standard deviation (σ), or discrimination threshold. In the unisensory conditions, the PSE was expected in the middle point of the temporal interval presented (around 500 ms, at the middle of the stimulus duration of 1,000 ms) suggesting no bias in the estimation. In the conflictual conditions, the point of subjective equality (PSE) producing separate estimates of the temporal bisection and precision thresholds for each conflictual condition were estimated. This condition is included to quantify the importance weighting of each sense when a conflict between senses is present. Given the presence of the conflict in the bimodal trials, in contrast to the unisensory condition, we might expect that the position of the PSE will not be centred at the middle of the interval because of these conflicts between stimuli. The bias for each conflictual condition was considered as the shift between the PSE of the conflictual condition and the PSE of the non‐conflictual condition. Since the conflict did not affect the threshold estimate (see Figure 4), all conflictual conditions were pooled to obtain the two‐cue threshold estimates. Both unimodal and bimodal (conflictual or not) audio and visual thresholds and PSEs were compared with the prediction of the Bayesian optimal‐integration model. Standard errors for the PSE and for the threshold estimates were obtained by bootstrapping (Efron & Tibshirani, 1993). One hundred bootstrap iterations were used and the standard error was the standard deviation of the bootstrap distribution.

### 2.5 Bayesian predictions

One of the earliest studies to investigate the capacity to integrate redundant information from multiple senses was done by Ernst and Banks (Ernst & Banks, 2002) who studied the integration of visual and haptic size estimates by human adults. Their results were consistent with a simple but powerful model proposing that visual and haptic input are combined in an optimal fashion that maximizes the precision of the final estimate. Their maximum likelihood estimate (MLE) model combines sensory information by summing the independent estimates from each modality, after weighting the estimates by their reliability, considered inversely proportional to the variance of the underlying noise distribution. The MLE calculation assumes that the optimal bimodal estimate of PSE ( ${\hat{S}}_{\text{VA}}$ ) is given by the weighted sum of the independent audio and visual estimates ( ${\hat{S}}_{V}$ and ${\hat{S}}_{A}$ ).

(1) $${\hat{S}}_{\text{VA}}=w_{V}{\hat{S}}_{V}+w_{A}{\hat{S}}_{A}$$

where weights $w_{V}$ and $w_{A}$ sum to unity and are inversely proportional to the variance (σ^2^) of the underlying noise distribution, assessed from the standard deviation σ of the Gaussian fit of the psychometric functions for visual and audio judgements:

(2) $$w_{V}=\sigma_{A}^{2}/\left(\sigma_{A}^{2}+\sigma_{V}^{2}\right),\;\;\;w_{A}=\sigma_{V}^{2}/\left(\sigma_{A}^{2}+\sigma_{V}^{2}\right)$$

The MLE prediction for the visual–audio threshold ( $\sigma_{\text{VA}}$ ) is given by:

(3) $$\sigma_{\text{VA}}^{2}=\frac{\sigma_{V}^{2}\sigma_{A}^{2}}{\sigma_{V}^{2}+\sigma_{A}^{2}}\leq \min\left(\sigma_{V}^{2},\sigma_{A}^{2}\right)$$

where $\sigma_{V}$ and $\sigma_{A}$ are the visual and audio unimodal thresholds. The improvement is greatest (factor of $\sqrt{2}$ ) when $\sigma_{V}=\sigma_{A}$ .

This model has been very successful in predicting human multimodal integration in adults for various tasks, including visual–haptic size judgements (Ernst & Banks, 2002), audio and visual position judgements (Alais & Burr, 2004) and visual–tactile integration of a sequence of events (Bresciani & Ernst, 2007). In children, this model can predict child behaviour only after 8–10 years of age, well after the maturation of individual senses (Gori et al., 2008; Nardini, Jones, Jones, Bedford, & Braddick, 2008; Nardini, Bedford, Bedford, & Mareschal, 2010; Gori et al., 2012; Gori et al., 2012; Gori et al., 2012; Nardini, Begus, Begus, & Mareschal, 2013).

## 3 RESULTS

**FIGURE 2:**
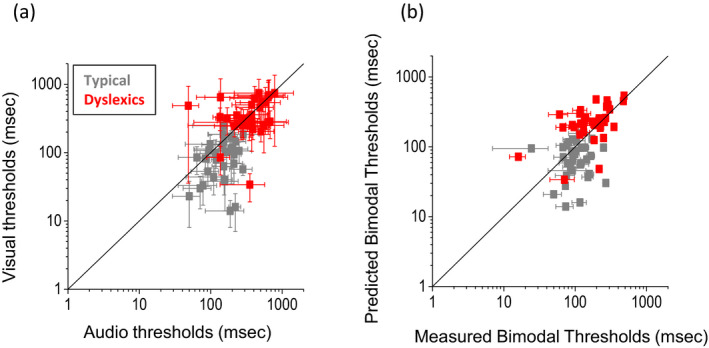
Individual thresholds for typical and dyslexic children. (a) Individual visual thresholds against auditory thresholds for the typical group in grey and for the dyslexia group in red. (b) Individual bimodal thresholds against Bayesian model predictions for the typical group in grey and for the dyslexia group in red. Threshold is estimated as sigma from the psychometric function. Error bars represent the standard deviation of the individual, estimated by bootstrap

Figure 2 reports the individual unisensory and multisensory thresholds (given by the standard deviation, see method section for more details) for the group of dyslexic (red dots) and typical (grey dots) children. On the left, unisensory visual thresholds are represented against unisensory audio thresholds. All dyslexic children (with the exception of two) show higher thresholds than the typical group. Moreover, audio and visual thresholds of the dyslexic group fall close to the equality line, indicating that their unisensory performance for the two unisensory tasks was similar. In contrast, the typical group shows unisensory thresholds falling mostly below the equality line, indicating better performance in the audio than in the visual task prediction, in agreement with Gori et al. (2012). On the right, multisensory thresholds are plotted against the thresholds predicted by the Bayesian model (calculated with Equation 3). In this case, while the thresholds of the dyslexic group fall close to the equality line (red dots), most of those of the typical group fall below to the equality line. This indicates that dyslexic children integrate in a predicted way while the typical group has multisensory thresholds higher than the Bayesian prediction, in agreement with Gori et al. (2012), indicating no Bayesian integration. Indeed, typical children, as previously demonstrated (Gori et al., 2012), showed no Bayesian integration for this task with audio dominance for these audio and visual temporal estimations.

**FIGURE 3:**
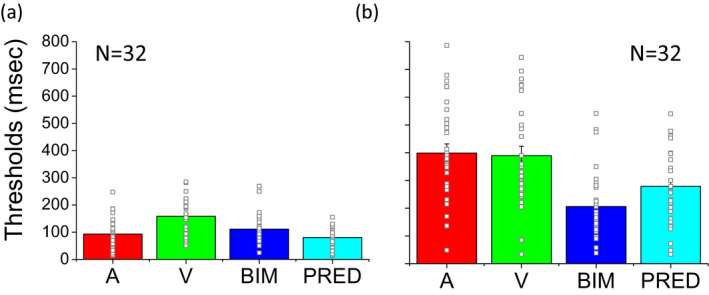
Average of unisensory visual, audio and bimodal thresholds. Average thresholds: visual, red; audio, green; audio and visual, blue; Bayesian prediction, light blue. (a) Unisensory and multisensory average thresholds and individual data for the typical group. (b) Unisensory and multisensory average thresholds and individual data for the group of dyslexic children. Error bars represent the standard error of the group

The same results can be observed in the average thresholds presented in Figure 3. A two‐way 2 × 3 ANOVA was performed for the evaluation of unisensory and multisensory thresholds considering sensory condition (visual, auditory and bimodal) as within‐participant factor and group (dyslexic and typical) as a between‐participant factor. Statistical analysis (ANOVA) revealed a very large group (*F*(1,62) = 76.9, *p* < .001, η_G_^2^ = 0.43) and sensory condition (*F*(2,124) = 23.1, *p* < .001, η_G_ = 0.13) effect as an interaction between group and sensory condition *F*(2,124) = 16.2, *p* < .001, η_G_^2^ = 0.1.

Typical children (Figure 3, left) have unisensory and multisensory thresholds that are significantly lower than the dyslexic group (Figure 3, right, two‐tailed *t* test, for visual condition *t*(62) = −6.94, *p* < .001, *d* = 1.74; for audio condition *t*(62) = −8.39, *p* < .001, *d* = 2.10; for bimodal condition *t*(62) = −4.46, *p* < .001, *d* = 1.11).

Auditory thresholds of the typical group are significantly lower than the visual thresholds (two‐tailed *t* test, *t*(31) = 5.09, *p* < .001, *d* = 0.9) and bimodal thresholds higher than predicted (*t* test, *t*(31) = 3.54, *p* < .005, *d* = 0.62).

**FIGURE 4:**
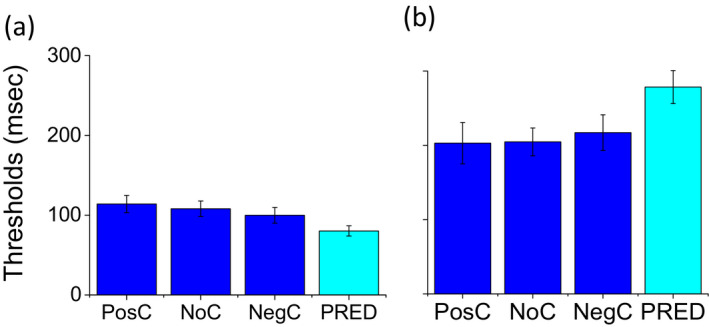
Average of unisensory visual and audio and bimodal thresholds and conflicts. Bimodal thresholds for the three different conflicts: positive conflicts, no conflicts and negative conflicts compared to the Bayesian prediction. (a) typical participants. (b) dyslexic participants. Error bars represent the standard error of the group

Contrary to this, auditory and visual thresholds of the dyslexic group did not significantly differ (two‐tailed *t* test, *t*(31) = 0.27, *p* = .79, *d* = 0.05) and the improvement due to multisensory audio and visual integration was well predicted by the Bayesian model (two‐tailed *t* test, *t*(31) = −1.27, *p* = .24, *d* = 0.22). This result suggests that unisensory auditory and visual thresholds of dyslexic children are worse than those of typical children. Multisensory thresholds were also worse in dyslexic than in typical children, but in dyslexic children, these were well predicted by the Bayesian model while in typical children they were not. The strong test of optimal integration is an improvement in bimodal thresholds (given by the standard deviation of the cumulative Gaussian fits). Data show that this improvement was present in the dyslexic and not in the typical children. All conflict conditions were merged to obtain the two‐cue threshold estimates in Figure 3. In Figure 4, to see the contribution of each conflictual cue on multisensory integration, we analysed the three conflictual conditions separately.

A two‐way 2 × 3 ANOVA was performed to evaluate the responses of the two groups in the conflictual conditions with conflicts (positive, neutral and negative) as the within‐participant factor and group (dyslexic and typical) as between‐participant factor. Statistical analysis (ANOVA) revealed a group (*F*(1,62) = 22.1, *p* < .001, η_G_^2^ = 0.19) but not a sensory condition (*F*(2,124) = 0.38, *p* = .69, η_G_^2^ = 0.002) effect. No effect was found in the interaction between group and sensory condition (*F*(2,124) = 1.641, *p* < .2, η_G_^2^ = 0.009).

Bimodal thresholds in all three of the conflictual conditions (positive conflict: where visual was presented 100 ms after audio, no conflict: where visual and audio stimuli were presented simultaneously, and negative conflict: where audio was presented 100 ms after vision) were not significantly different from the Bayesian prediction in dyslexic children (two‐tailed paired *t* test, *t*(31) = −1.39, *p* = .17, *d* = 0.25 for positive conflict, *t*(31) = −1.75, *p* = .09, *d* = 0.30 for no conflict and *t*(31) = 0.07, *p* = .94, *d* = 0.01 for negative conflict) and significantly different in typical children (two‐tailed paired *t* test, *t*(31) = 3.30, *p* < .005, *d* = 0.58 for positive conflict, *t* test, *t*(31) = 3.18, *p* < .005, *d* = 0.56 for no conflict and *t* test, *t*(31) = 2.27, *p* < .05, *d* = 0.40 for negative conflict). These results suggest that multisensory improvement in dyslexic children was present for all conflictual conditions considered.

**FIGURE 5:**
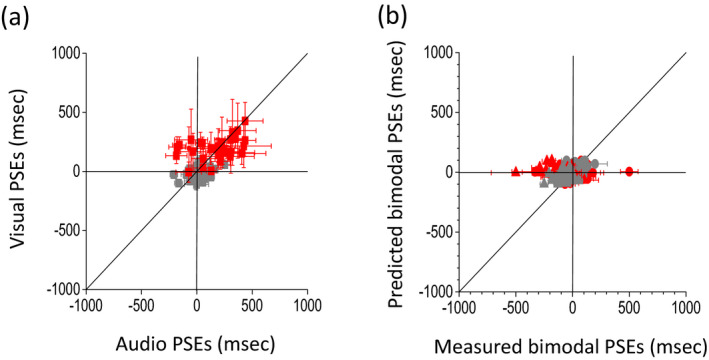
Individual PSEs for the two groups. (a) Individual visual PSEs against audio PSEs for the typical group in grey and for the dyslexia group in red. (b) Individual bimodal PSEs for the three conflict conditions in different symbols against Bayesian prediction for the typical group in grey and for the dyslexia group in red. PSE is estimated from the mean of the psychometric function. Error bars represent the standard deviation of the individual subject estimated by bootstrap

Figure 5 reports the PSEs (point of subjective equality, given by the mean of the error functions: the 50% point, see method section for more details). A two‐way 2 × 3 ANOVA was performed for the evaluation of unisensory and multisensory PSEs with sensory condition (visual, auditory and bimodal) as a within‐participant factor and group (dyslexic and typical) as a between‐participant factor. Statistical analysis (ANOVA) revealed large group (*F*(1,62) = 43.08, *p* < .001, η_G_^2^ = 0.25) and sensory condition (*F*(2,124) = 4.57, *p* < .05, η_G_^2^ = 0.04) effects as an interaction between group and sensory condition (*F*(2,124) = 9.96, *p* < .001, η_G_^2^ = 0.08). Similar to the audio and visual unisensory thresholds, unisensory PSEs (Figure 5a) also differ for the two groups of participants (two‐tailed *t* test, *t*(62) = −7.49, *p* < .001, *d* = 1.08).

**FIGURE 6:**
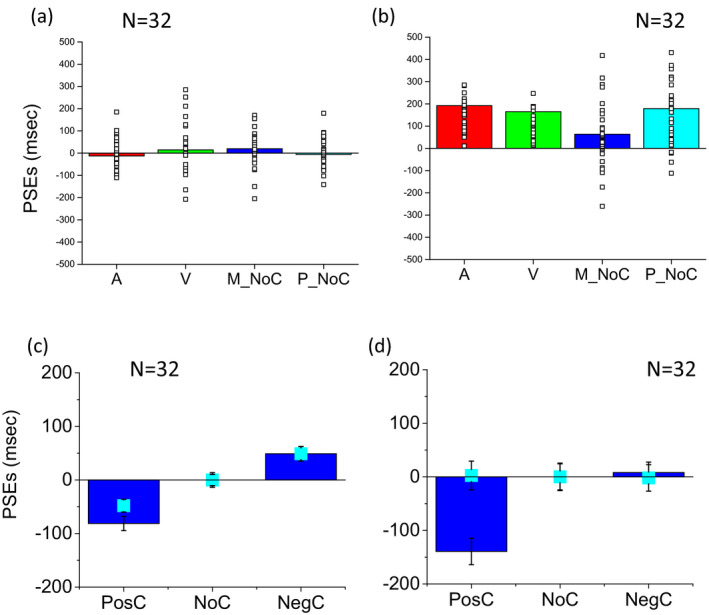
Average of unisensory visual, audio and conflictual and non‐conflictual bimodal PSEs. Average PSEs: visual, red; audio, green; audio–visual, blue; Bayesian prediction, light blue. (a) Unisensory and multisensory average PSEs and individual data for the typical group. (b) Unisensory and multisensory average PSEs and individual data for the group of dyslexics children. (c) Bimodal PSEs for the three different conflicts (blue bars): positive conflicts, no conflicts and negative conflicts compared to the Bayesian prediction (light blue dots) for typical participants. (d) Bimodal PSEs for the three different conflicts for dyslexic participants. Error bars represent the standard error of the group

Figure 6 reports the average PSEs and individual data for typical and dyslexic children directly comparing unisensory, multisensory conditions and model predictions. Figure 6a,b shows the individual and average PSEs for the audio, visual and bimodal non‐conflictual condition with the model prediction. Figure 6c,d shows PSEs (normalized to the no bias condition) for typical and dyslexic children for all the bimodal conditons. Dyslexic children have significantly more positive audio (two‐tailed *t* test, *t*(62) = −10.83, *p* < .001, *d* = 2.71) and visual (two‐tailed *t* test, *t*(62) = −4.05, *p* < .001, *d* = 1.01) PSEs than the typical group. More positive PSEs in visual and auditory conditions means that dyslexic children, but not typical participants, tend to compress the first interval and to expand the second interval to perceive them at the same distance from the two temporal extremes of the stimulus. This result suggests that unisensory estimations of dyslexic children are more biased than those of typical children. In particular, dyslexic children in the unimodal condition tend to judge the second stimulus as closer in time to the first stimulus up to approximately halfway into the interval that was actually closer to the third stimulus. Specifically, dyslexic children on average needed at least a 750 ms gap between the first and second stimuli, and a less than a 175 ms gap between the end of the second and start of the third stimuli, before they became more likely to judge the second interval as smallest. In contrast, bimodal PSEs were not significantly different between the two groups (two‐tailed *t* test, *t*(62) = −1.50, *p* = .41, *d* = 0.38). In Figure 6c,d for the bimodal conditions, the PSEs for constant errors in bias were adjusted by subtracting from each conflictual PSE the PSE obtained in the non‐conflictual condition for each participant. The same correction was applied to the prediction of the Bayesian model. An analysis of bimodal PSEs considering the three conflicts shows a good prediction for both typical and dyslexic children for the negative conflict (when vision precedes audio information by 100 ms; typical two‐tailed *t* test, *t*(31) = 0.43, *p* = .67, *d* = 0.08; dyslexic two‐tailed *t* test, *t*(31) = 0.08, *p* = .08, *d* = 0.25). A poor prediction was only observed in both typical and dyslexic children in the positive conflicts (when vision was presented after the audio information by 100 ms; typical two‐tailed *t* test, *t*(31) = −2.92, *p* < .01, *d* = 0.52, dyslexic two‐tailed *t* test, *t*(31) = −3.43, *p* < .005, *d* = 0.61). Statistical analysis (ANOVA) revealed no effect of group (*F*(1,62) = 0.29, *p* = .6, η_G_^2^ = 0.003). An effect of group for sensory condition was observed (*F*(2,124) = 45.962, *p* < .01, η_G_^2^ = 0.25). An absent effect of interaction between group and sensory condition was observed (*F*(2, 124) = 1.9, *p* = .15, η_G_^2^ = 0.01).

Given the wide range in age, we checked whether unimodal and bimodal thresholds differed between younger (7–9 years of age) and older children (10–14 years) of dyslexic and typical children. No significant difference was observed for the two age ranges in the typical group (audio *t*(30) = 0.35, *p* = .73, *d* = 0.12; visual *t*(30) = 0.72, *p* = .48, *d* = 0.26; bimodal *t*(30) = −0.18, *p* = .86, *d* = 0.06) or dyslexic group for the visual modality (*t*(30) = 0.67, *p* = .51, *d* = 0.24). Significant difference was observed for audio and bimodal threshold improvement in the dyslexic group between the younger group (7–9 of age) and the older group (10–14 years) (audio *t*(30) = −2.41, *p* < .05, *d* = 0.86 and bimodal *t*(31) = −2.59, *p* < .05, *d* = 0.92). This suggest that in dyslexic children there is a developmental trend affecting audio and bimodal ability during the age range considered.

Given the presence of dyslexic children speaking two different languages, we checked whether unimodal and bimodal thresholds differed between the Italian and Polish groups. No significant difference was observed between the two groups (audio: *t*(30) = −0.89, *p* = .38, *d* = 0.24; visual: *t*(30) = 0.08, *p* = .94, *d* = 0.12; bimodal: *t*(30) = −1.44, *p* = .16, *d* = 0.92). This suggests that the deficit was not specific for one language and supports the idea that the deficit was not related to the different learning strategies for reading learning in the two countries.

**FIGURE 7:**
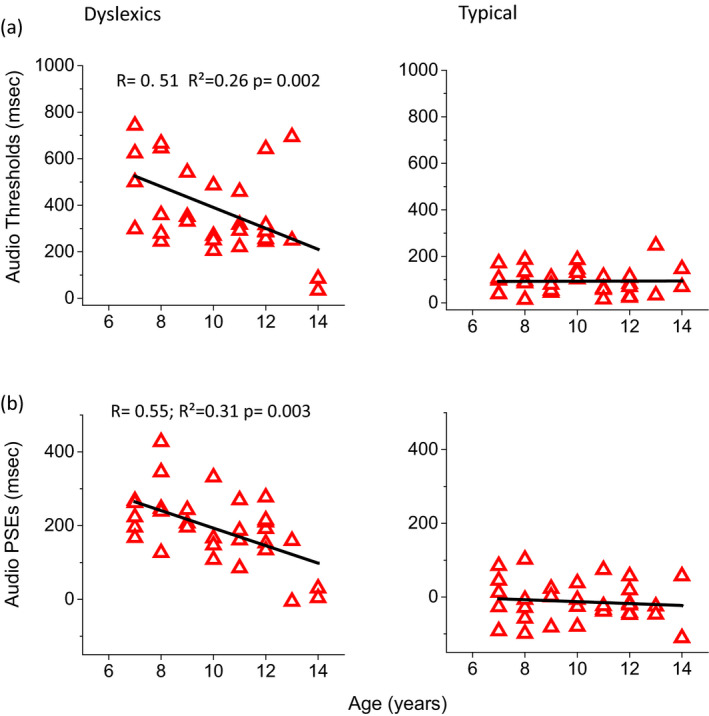
Audio thresholds and PSEs as a function of years of schooling. (a) Audio thresholds as a function of years of schooling in dyslexic and typical children. (b) Audio PSEs as a function of years of schooling in dyslexic and typical children. Continuous lines represent linear regression of the data

Figure 7 shows the correlation between age, audio thresholds and PSEs in dyslexic and typical children (*R* = .51; *R* ^2^ = .26; *p* = .0017; rho = −0.507, 95% CI [−0.73, −0.19]) and for audio PSEs (*R* = .55; *R* ^2^ = .31; *p* = .003 rho = −0.502, 95% CI [−0.72, −0.19]). No correlation was observed in typical children for both audio thresholds (*R* = .01; *R* ^2^ < .001; *p* = .95; rho = −0.029, 95% CI [−0.37, 0.32] calculated with Spearman rank estimator) and for audio PSEs (*R* = .01; *R* ^2^ = .001, *p* = .56, rho = −0.098 95% CI [−0.43, 0.25]). The correlation between dyslexic and typical children was significantly different for both audio thresholds (*z* = 3.0; two‐tailed *z*‐test *p* = .001) and audio PSEs (*z* = 3.25; two‐tailed *z*‐test *p* = .001). Similarly, a correlation was observed for bimodal thresholds and age in dyslexic children (data not shown, *R* = .42; *R* ^2^ = .18; *p* = .01). No correlation was observed for typical children. No correlation was observed for visual thresholds and PSEs in either groups.

**FIGURE 8:**
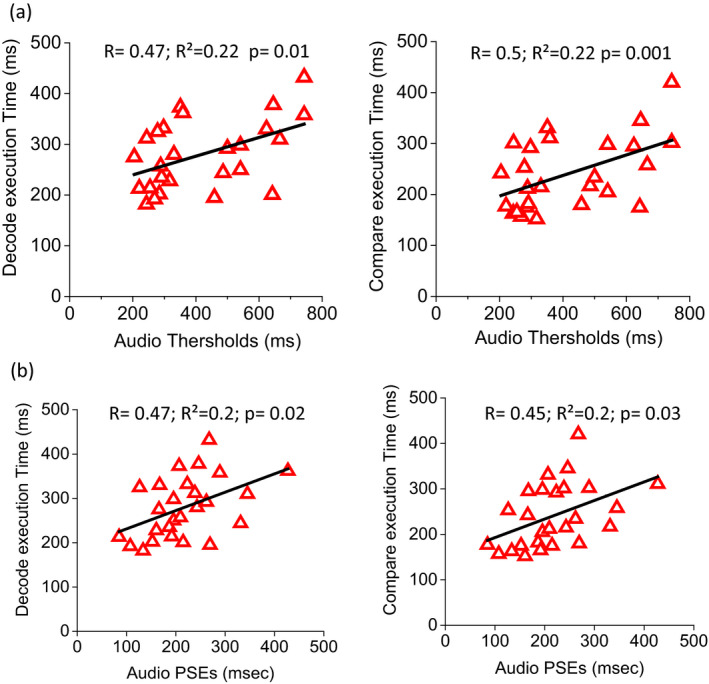
Bimodal thresholds and PSEs as a function of reading capabilities. A. Execution time to compare and the decode Prolexia test as a function of audio thresholds. B. Execution time to compare and the decode Prolexia test as a function of audio PSEs. Continuous lines represent linear regression of the data

A correlation was also observed between audio thresholds and reading capabilities in dyslexic children measured with the Prolexia test (Ober et al., 1998). Only 26 of 32 dyslexic children completed this test and were considered in the analysis (see method section for more details; Figure 8): greater audio deficit correlates with lower reading performance. Audio thresholds (Figure 8a) correlate with decode execution time (*R* = .47; *R* ^2^ = .22; *p* = .01, rho = 0.43 95% CI [0.05, 0.70])) and compare execution time (*R* = .5; *R* ^2^ = .22; *p* = .001, rho = 0.51 95% CI [0.15, 0.75]. Similarly, audio PSEs (Figure 8b) correlate with decode execution time (*R* = .47; *R* ^2^ = .2; *p* = .02; rho = 0.48 95% CI [0.11, 0.73) and compare execution time (*R* = .45; *R* ^2^ = .2; *p* = .03; rho = 0.49 95% CI [0.13, 0.74]). These results suggest that there is an association between temporal skills of dyslexic children and their reading abilities.

## 4 DISCUSSION

**The first insight** of this study is the recognition of a gross deficit in audio and visual temporal bisection in dyslexic children: unisensory audio and visual bisection temporal thresholds were higher in dyslexic than in typical children (Figures 2 and 3). A decision in a bisection task is not based on an instantaneous estimate but requires participants to encode the temporal sequence of three sounds, remember them and compare their remembered temporal sequence (see Figure 1 for the stimulus structure). It requires a representation of time that must remain in the participant's memory for the duration of the task (one second) and a temporal map. This task is a simple perceptual task that requires a representation of time and its metric knowledge, and relies heavily on a temporal metric representation map. We previously used this stimulus to study temporal representation in typical children (Gori et al., 2012). Results suggest that already at 6 years of age, typical children have no difficulty in understanding the task, and around 10 years of age their thresholds were close to those of adults (Gori et al., 2012). With the same temporal bisection task, we have also recently highlighted a deficit in temporal representation in deaf children (Gori et al., 2017). Here, we show for the first time that this temporal task is complex for dyslexic children, who show less precise and distorted estimations. The importance of word metric structure and, specifically, rise‐time perception for speech processing has been previously stressed by other researchers (e.g. Goswami et al., 2002). Distorted audio and visual temporal representation can interfere with the temporal representation of visual and audio stimuli in dyslexic children, necessary to couple and mapping graphene with a phoneme.

The deficit observed in this work is in agreement with previous work showing that children with dyslexia perform poorly on tasks of rhythmic perception and perception of a musical meter (Huss, Verney, Verney, Fosker, Mead, & Goswami, 2011). Our task can be considered a rhythm task since the temporal intervals between three sounds need to be remembered and interpreted as a unique stimulus. In both unisensory and multisensory tasks, temporal bisection thresholds were higher in dyslexic than in typical children, suggesting low precision of dyslexic children in temporal rhythm perception. The impairment in the unisensory audio and visual processing is also in agreement with other previous studies showing a deficit in the auditory and visual domain of dyslexic children. For example, dyslexic people have difficulty in discriminating dynamic visual stimuli (e.g. Stein & Walsh, 1997). Dyslexic children also fare less well than typical participants in several auditory tasks that require the perception of brief or rapid speech and non‐speech sounds (Tallal, Miller, Miller, & Fitch, 1993). Interestingly, it has been reported that dyslexic children have difficulty in processing stimuli occurring at a frequency on the order of 2–10 cycles per second, which is the approximate frequency of syllables (Goswami et al., 2002; Goswami, Fosker, Fosker, Huss, Mead, & Szucs, 2011; Goswami, Huss, Huss, Mead, Fosker, & Verney, 2013). The recent linguistic analysis suggested that stressed syllables occur approximately every 500 ms (2 Hz) (Arvaniti, 2009) and impairments in perceiving syllable stress have been recently found in dyslexia (Leong & Goswami, 2014). Our stimuli are within this temporal range requiring specific attention every 500 ms (2 Hz) and might be associated with a similar processing.

Results on unisensory PSEs suggest that unisensory audio and visual estimations of the middle point between two lateral stimuli differ in dyslexic children compared to in typical children. Dyslexic children have significantly more positive audio and visual PSEs than the typical group, meaning that they tend to compress the first interval and to expand the second interval to perceive them at the same distance from the two temporal extremes of the stimulus. We might speculate that this compression and expansion of the two intervals, together with the lower precision in the audio and visual estimations discussed above, could hamper the correct mapping of phonemes and syllables into words. Importantly, the presence of the same deficit in two languages (of Italian and Polish children) suggests that the present deficit is associated with a basic sensory impairment (e.g. in terms of regularity of grapheme–phoneme mapping) that is generalized among different languages.

Most studies on dyslexia have used speech sound–grapheme combinations or audio and visual speech stimuli (Froyen et al., 2008; Froyen et al., 2009, 2011; Mittag et al., 2013) to show deficits in print–speech integration (e.g. (Kronschnabel, Brem, Brem, Maurer, & Brandeis, 2014). Letter–speech sound stimuli are complex, both for the auditory and visual systems and for their integration processing. Some authors have suggested that different patterns of integration between dyslexic and typical individuals could be observed for basic multisensory configurations (as suggested by Molholm et al., 2002; Brandwein et al., 2011; Brandwein et al., 2013; Hahn, Foxe, Foxe, & Molholm, 2014). In agreement with this idea, here, using simple not‐linguistic stimuli, we showed that dyslexic children processed differently than typical children multisensory signals. Indeed, **the second insight of this work** is that dyslexic children, unlike typical children, show an optimal multisensory integration for temporal bisection, well predicted by the Bayesian model (Figures 3 and 4). Our multisensory results were modelled using the Bayesian approach (Ernst & Banks, 2002; Alais & Burr, 2004; Landy et al., 2011). The Bayesian approach predicts that different sensory inputs are combined after weighting unisensory visual and auditory signals for reliability. This model has been shown to be a powerful method in predicting multisensory integration in many tasks (Ernst & Banks, 2002; Alais & Burr, 2004; Landy et al., 2011). In children, reliability‐based multisensory integration seems to develop late, after 8–10 years of age: before then, one sense (such as audio or haptic) dominates the other (such as vision) (Gori et al., 2008; Gori et al., 2012; Gori et al., 2012). On the other hand, for the temporal bisection task, used in this work, multisensory integration in typical children and adults is not evident showing auditory dominance in multisensory estimation at all ages (Gori et al., 2012). We think that the auditory dominance in typical children observed in this work and in the previous work (Gori et al., 2012) can reflect a process of cross‐sensory calibration in which the auditory system could be used to calibrate the visual sense of time since it is the most accurate sense for temporal judgements. This result is in agreement with many experiments performed with adults that show a dominant role of the auditory system for time (Gebhard & Mowbray, 1959; Sekuler & Sekuler, 1999; Shams, Kamitani, Kamitani, & Shimojo, 2000; Shams, Kamitani, Kamitani, Thompson, & Shimojo, 2001; Berger, Martelli, Martelli, & Pelli, 2003; Burr, Banks, Banks, & Morrone, 2009). Why the auditory dominance of both PSEs and bimodal thresholds persists into adulthood is not clear. A possible explanation is that for this kind of task the cross‐sensory calibration process is still occurring since audition is too accurate with respect to the visual modality, and the precision of the visual system for this kind of task prevents the transition from unisensory dominance to multisensory integration. This idea is in agreement with another recent findings in children with Cochlear Implants (Gori et al., 2017) who show lower auditory thresholds and show optimal integration for audio and visual temporal signals. Similar to children with cochlear implants, our results suggest that dyslexic children, unlike typical children, show strong multisensory integration, with bimodal thresholds well predicted by the Bayesian model. A possible speculation is that the auditory system dominates the visual one when it is highly more precise, as in the case of typical children. On the other hand, when the visual and the auditory unisensory thresholds are similar, such as in the case of children with dyslexia, auditory dominance does not occur and vision might be used as a support to calibrate time. Audio–visual integration may be fundamental to restoring many important temporal properties when audio temporal skills are impaired and typical audio dominance cannot occur. A speculation is that vision might be used as a support to calibrate time when audition is impaired (in agreement with, e.g., Tyler, Fryauf‐Bertschy, Fryauf‐Bertschy, Gantz, Kelsay, & Woodworth, 1997; Giraud, Price, Price, Graham, Truy, & Frackowiak, 2001; Green, Julyan, Julyan, Hastings, & Ramsden, 2005; Doucet, Bergeron, Bergeron, Lassonde, Ferron, & Lepore, 2006; Gori et al., 2017).

Since dyslexic children also show higher audio and visual temporal thresholds in comparison with the typical group, although well predicted by the Bayesian model, the precision in the multisensory condition was lower (higher threshold) in the group of dyslexic children than in the typical group. It implies that the gain obtained by the presence of multiple cues in dyslexic children is not enough to reach typical levels. On the other hand, our results suggest that these lower multisensory performances derive from lower precision in the unisensory inputs rather than from an impairment in the multisensory processing per se.

In dyslexic children, good multisensory integration, in terms of improvement of precision on multisensory compared to unisensory conditions, was observed for both conflictual and non‐conflictual multisensory audio and visual information presented, namely when vision precedes the audio signal by 100 ms, when the audio signal precedes the visual signal by 100 ms and when signals were presented simultaneously (Figure 4). However, no increment in multisensory precision suggesting good integration was observed in the typical group for any of the multisensory conditions tested. This suggest that while dyslexic children benefit from the presence of multiple audio and visual signals, improving their precision in the estimation when both signals are available, the same is not true for typical children. A different pattern is observed for the predicted PSEs. Indeed, analysis of PSEs shows that both typical and dyslexic groups integrated as predicted no and negative conflicts, while both show a bias, stronger in dyslexic children in the multisensory positive condition. When the visual information is presented 100 ms after the auditory stimulus, the visual signal is perceived simultaneous to the audio signal only when it is present a 150 ms delay in dyslexic (and less than 100 ms in typical) between the visual and auditory stimuli. Previous works have hypothesized that anomalies in the length of the window of integration in dyslexic children might contribute to reading difficulty in dyslexia (Blau, van Atteveldt, van Atteveldt, Ekkebus, Goebel, & Blomert, 2009; Blau et al., 2010; Mittag et al., 2013). The temporal window of integration refers to the window within which multisensory inputs tend to be integrated into one perceptual unit (King & Palmer, 1985; Meredith, Nemitz, Nemitz, & Stein, 1987; Spence & Squire, 2003). The temporal window is important for the proper binding of speech sounds to print when learning to read. A large or overly variable window might indeed lead to greater errors in the accurate pairing of orthography and speech sounds in dyslexic individuals (Blau et al., 2009; Blau et al., 2010; Mittag et al., 2013). Our result on PSE is in agreement with previous works showing that dyslexic children have good integration only at the later timeframe when there was a 200 ms delay between the visual and auditory stimuli (Froyen et al., 2011). This temporal window difference in dyslexia could contribute to difficulties in learning grapheme–phoneme associations. On the other hand, since we found the same bias for both dyslexic (of about 150 ms) and typical children (of about 80 ms), we note that this difference does not appear to be specific to dyslexic children and thus is unlikely to explain the deficit we observed, which was specific to dyslexic children.

Some studies have shown that multisensory integration skills vary with attentional processing (Sutton, Hakerem, Hakerem, Zubin, & Portnoy, 1961) and sometimes dyslexia is associated with attentional problems. Our bisection task requires memorization of three sounds in time and attentional load to evaluate the position in time of the second stimulus with respect to the other two. For this reason, another possible cause of the deficit observed here can be related to different attentional or working memory processes between typical and dyslexic children. In a recent study, Harrar et al. (2014) tested a non‐linguistic audio and visual task in dyslexic adults and observed dyslexic adults had difficulty shifting their attention between modalities by measuring their reaction times (RTs) to multisensory stimuli compared to predictions from Miller's race model (Miller, 1982, 1986). They suggested that dyslexic individuals distribute their cross‐modal attention resources differently from typical subjects, causing different patterns in multisensory responses compared to the typical group. The test performed in our work cannot directly evaluate whether this attentional processing influenced our multisensory task. Indeed, with our test, it was not possible, for example, to exclude that the pattern we observed was related to a response bias, namely the preference to say ‘the first interval’ and further tests will be necessary to exclude this possibility.

It has been also shown that dyslexic individuals have difficulty allocating their unisensory visual attention resources across space (Vidyasagar, 1999) and for this reason it is important to present aligned audio and visual stimuli (Spence, Nicholls, Nicholls, & Driver, 2001). Our audio and visual stimuli were co‐localized (audio and visual stimuli were superimposed) and we can rule out the deficit observed here as being due to a deficit of attentional distribution across modalities and across space. In our study, we also maintained a constant light sequence to facilitate the task in children. Similarly, we fixed the sequence of conditions, always presenting the unisensory stimuli before the multisensory stimuli. By maintaining the same sequence among different groups of children (typical and dyslexic in both countries), we expect that possible effects due to learning should be comparable among groups and do not affect the results. The different performance we observed in the two groups, with the typical group performing better in the unisensory conditions (presented before) than the multisensory ones and with the dyslexic group gaining more from the multisensory conditions than unisensory (presented before) suggests that different mechanisms are acting in the two groups.

Although the deficit we observed in unisensory and multisensory perceptions was stable among the group, it would be interesting in future studies to examine in more detail the relationships between performance in our tests and individual attentional skills of children. Finally, we can exclude IQ differences within the dyslexic group as the cause of different performances in our bisection tasks. Our group was composed of dyslexic children mostly with an IQ > 70 (with 19 out of 32 with an IQ > 80; Table 1). The IQ results were not correlated with the audio, visual or bimodal performances (*R* = .1, *p* = .6).

**The third insight** from this work is that the deficit in the audio bisection task correlates with age and with reading capability in dyslexic children but not in typical children. This suggests that the bisection deficit is associated with the experience of written language but only in dyslexic children, which show a specific deficit in reading. Contrary to other studies that have observed differences between dyslexic and typical children before learning to read (Saygin et al., 2013), we found differences between the dyslexic and typical group during and after learning to read. Our result is in agreement with a previous study (Flaugnacco et al., 2014) showing that level of performance on a metric of perception specifically predicted both reading speed and accuracy as well as phonological processing in dyslexic people. It is also in agreement with other works showing that dyslexic children have problems on grouping stimuli and on musical meter task and that rhythmic impairment is associated with their reading abilities (Goswami et al., 2011; Huss et al., 2011; Stefanics et al., 2011).

## 5 CONCLUSIONS

Numerous theories have been proposed to explain dyslexia (Morgan, 1896; Lovegrove, Bowling, Bowling, Badcock, & Blackwood, 1980; Tallal, 1980; Wagner, 1986; Galaburda, 1994; Eden, Stein, Stein, Wood, & Wood, 1995; Stein & Walsh, 1997; Frost, 1998; Snowling et al., 2000; Ramus, 2001; Stein, 2001; Valdois, Bosse, Bosse, & Tainturier, 2004; Vellutino et al., 2004; Heim et al., 2008; Ramus & Szenkovits, 2008; Kovelman et al., 2012; Hamalainen et al., 2013; Ramus et al., 2013; Hahn et al., 2014; Harrar et al., 2014). Our results suggest that dyslexic children have a severe impairment in the creation of unisensory visual and auditory temporal representations and show a compression and expansion of temporal intervals during interval segmentation.

The correlation between auditory temporal skills, age and reading ability supports the idea that correct temporal audio representation perception is fundamental to the reading process. Scientists have agreed that time perception is fundamental in audition (e.g. VanRullen, Zoefel, Zoefel, & Ilhan, 2014; Martin, Kosem, Kosem, & van Wassenhove, 2015; Desantis & Haggard, 2016). We recently showed that deaf individuals with hearing restored have lower audio and visual temporal performances in a temporal bisection task than typical children. More interestingly, we observed that lower level auditory temporal skills in deaf children with restored hearing were associated with lower language capabilities (Gori et al., 2017). We have suggested that this dominance, specific for the task performed, could reflect a process of cross‐sensory calibration in which the modality that is the most robust (not necessarily the most reliable) is the calibrator (Gori, Sandini, Sandini, Martinoli, & Burr, 2010, 2014; Sciutti, Burr, Burr, Saracco, Sandini, & Gori, 2014; Gori, 2015; Vercillo, Milne, Milne, Gori, & Goodale, 2015; Vercillo, Burr, Burr, & Gori, 2016). In particular, we have suggested that auditory experience during development is fundamental for temporal learning. Audio and visual multisensory integration and in particular auditory temporality is fundamental for efficient language acquisition (van Wassenhove, Grant, Grant, & Poeppel, 2005, 2007; Megevand, Molholm, Molholm, Nayak, & Foxe, 2013; van Wassenhove, 2013). Audio and visual temporal links that are established naturally (VanRullen et al., 2014; Martin et al., 2015; Desantis & Haggard, 2016) play a key part in the role of audition in language development (for a review, see Cardon, Campbell, Campbell, & Sharma, 2012). These observations support the idea that an audio rhythm deficit might affect the ability to correctly develop speech and reading skills. In agreement with this idea, recent studies have shown that individuals with dyslexia show a decreased activation in the auditory cortex and in the superior temporal sulcus compared to strong readers (Blau et al., 2009; Blau et al., 2010; Hahn et al., 2014). We might also speculate that, if auditory information is fundamental for the calibration of visual temporal processing, the deficit in the auditory system in dyslexia might affect the correct development of visual temporal perception, which was also impaired in our test.

Recently, results on rhythm impairment in dyslexia inspired the development of temporal training based on simple non‐linguistic stimuli (Kujala et al., 2001; Veuillet, Magnan, Magnan, Ecalle, Thai‐Van, & Collet, 2007; Ecalle, Magnan, Magnan, Bouchafa, & Gombert, 2009; Kast, Baschera, Baschera, Gross, Jancke, & Meyer, 2011). Reading improvement following this non‐linguistic training programme suggests a role for a more general multisensory processing deficit in dyslexia. In particular, recent studies suggest that music training based on rhythmic stimulation can help overcome dyslexia (Flaugnacco et al., 2014, 2015; Habib et al., 2016).

Semantically unrelated temporal stimuli, such as that presented here, might be useful for diagnostic purposes after systematic comparison with existing screening tests for dyslexia. Importantly, these tests could be adopted prior to reading acquisition, thus providing a tool for early intervention and rehabilitation.

## AUTHORS' CONTRIBUTION

MG, OAC, KMO and FT collected data, MG wrote the manuscript. MG and OAC participated in protocol definition and paper discussion.

## COMPETING FINANCIAL INTERESTS

The author(s) declare no competing financial interests.

## Supporting information

Supplementary Material

## Data Availability

Data sharing is not applicable to this article as no new data were created or analysed in this study.
