# Supplementary material for: Temporal representation impairment in developmental dyslexia for unisensory and multisensory stimuli
Source: Dev Sci. 2020 Jun 14;23(5):e12977. doi: 10.1111/desc.12977 (PMC7507191; doi:10.1111/desc.12977)

Typical

CEx1

OV

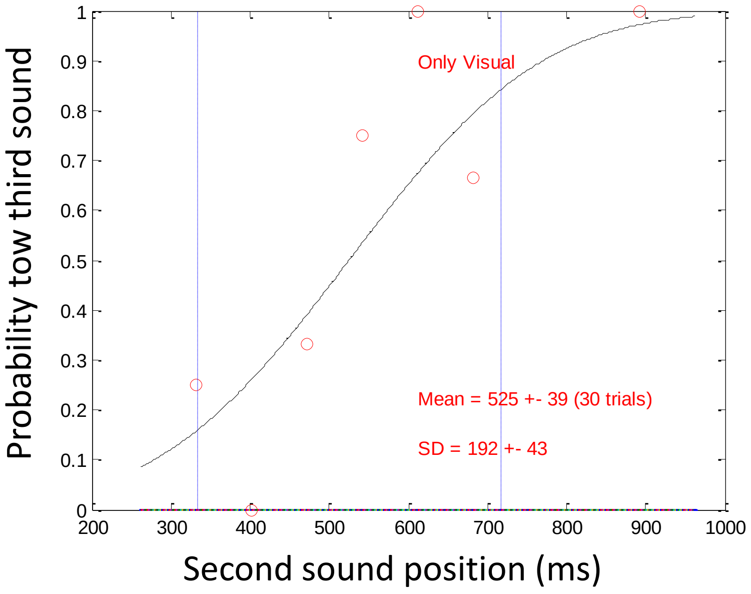

OA

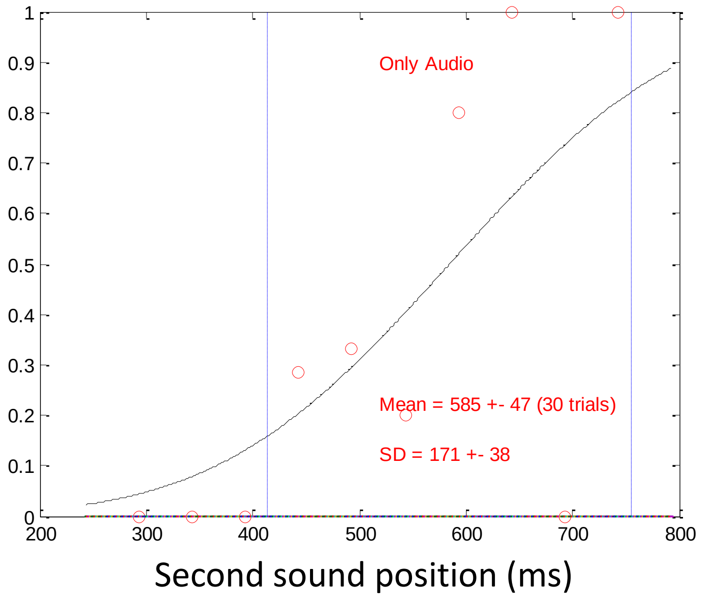

Predicted				
	PConf	NoConf	NConf	Beta Bim
	47	58	70	128

Bimodal

V=50,A=-50

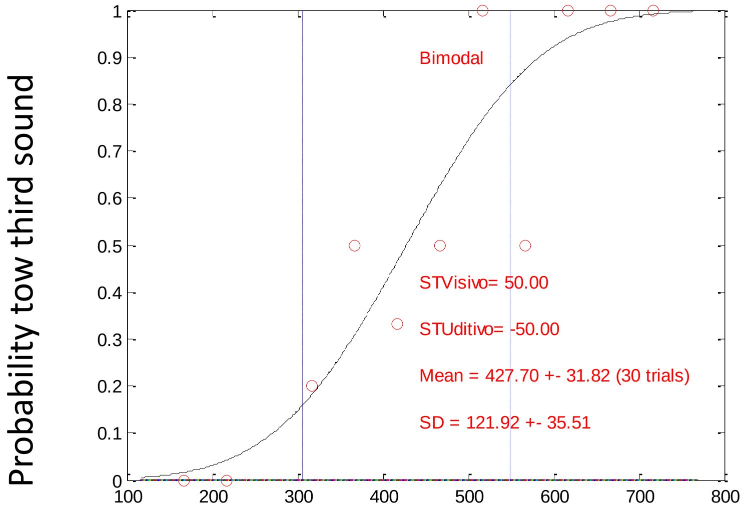

V=0,A=-0

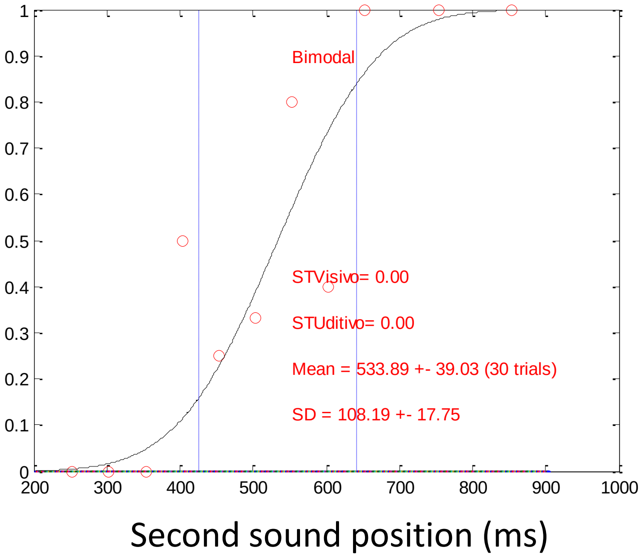

V=-50,A=50

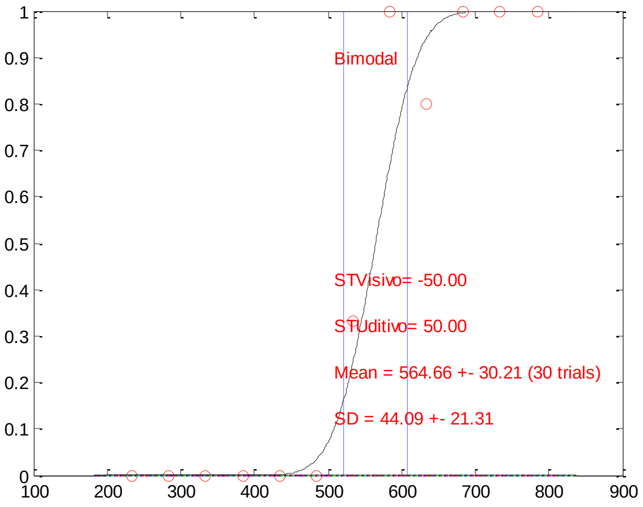

CEx2

OV

OA

Predicted				
	PConf	NoConf	NConf	Beta Bim
	-119	-103	-87	75

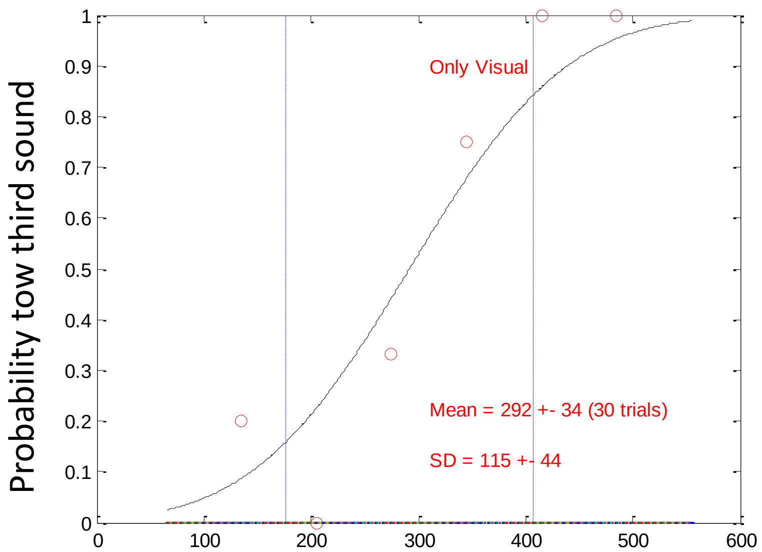

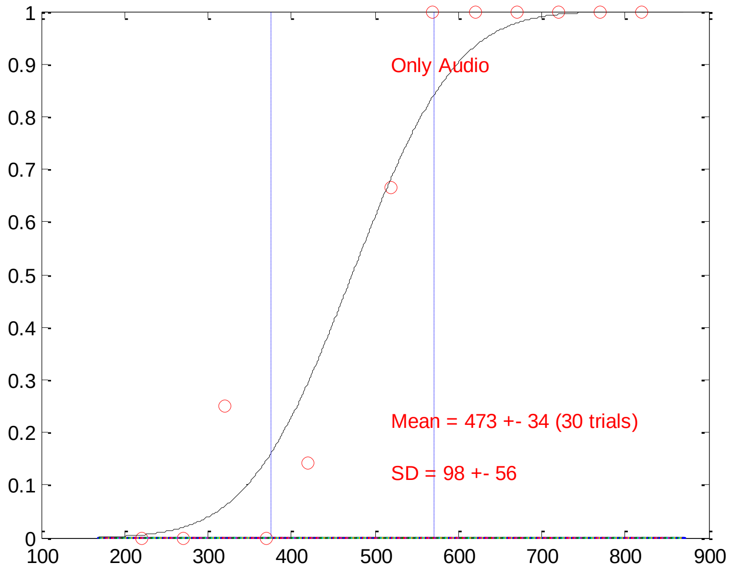

Second sound position (ms)

Bimodal

V=50,A=-50

V=0,A=-0

V=-50,A=50

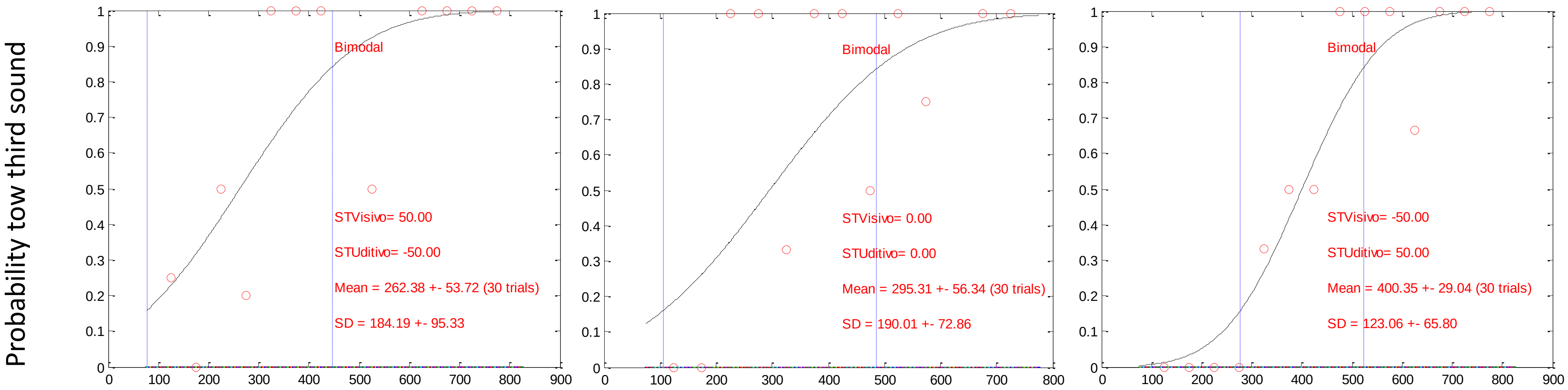

Second sound position (ms)

CEx3

Predicted				
	PConf	NoConf	NConf	Beta Bim
	-33	-61	-89	51

OV

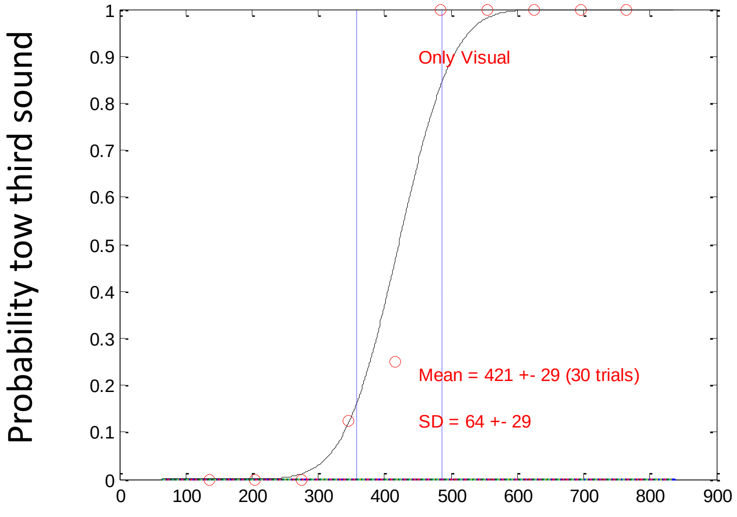

OA

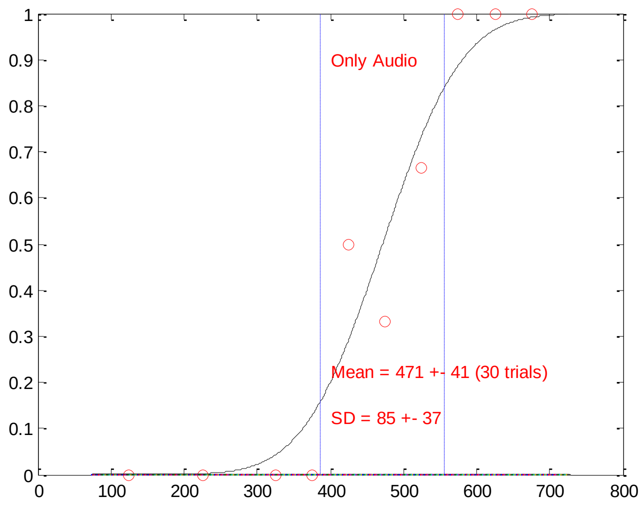

Second sound position (ms)

Bimodal

V=50,A=-50

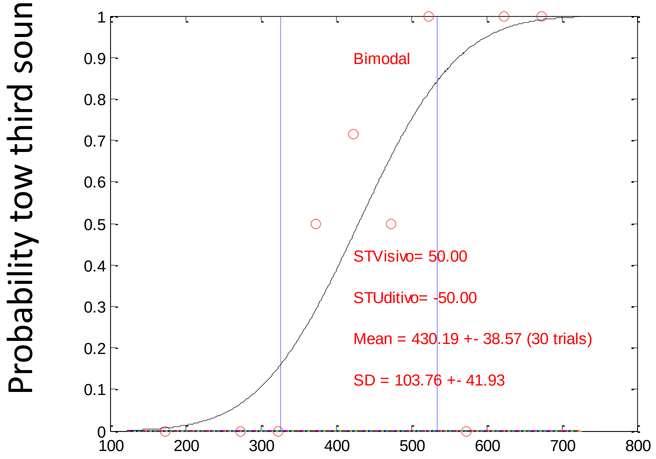

V=0,A=-0

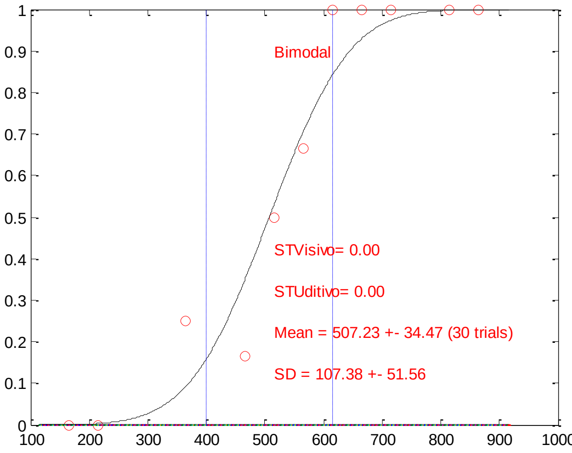

V=-50,A=50

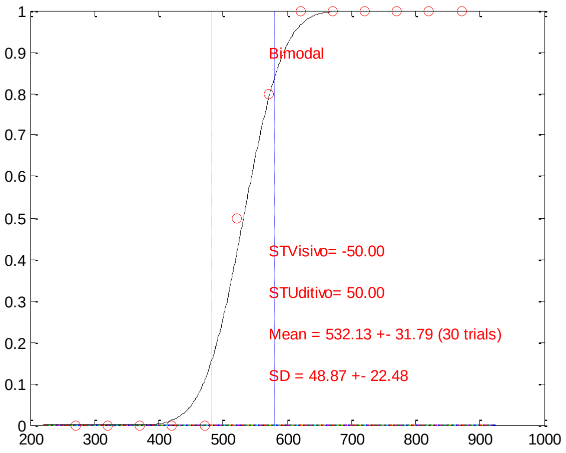

Second sound position (ms)

CEx4

Predicted				
	PConf	NoConf	NConf	Beta Bim
	-115	-76	-37	66

OV

OA

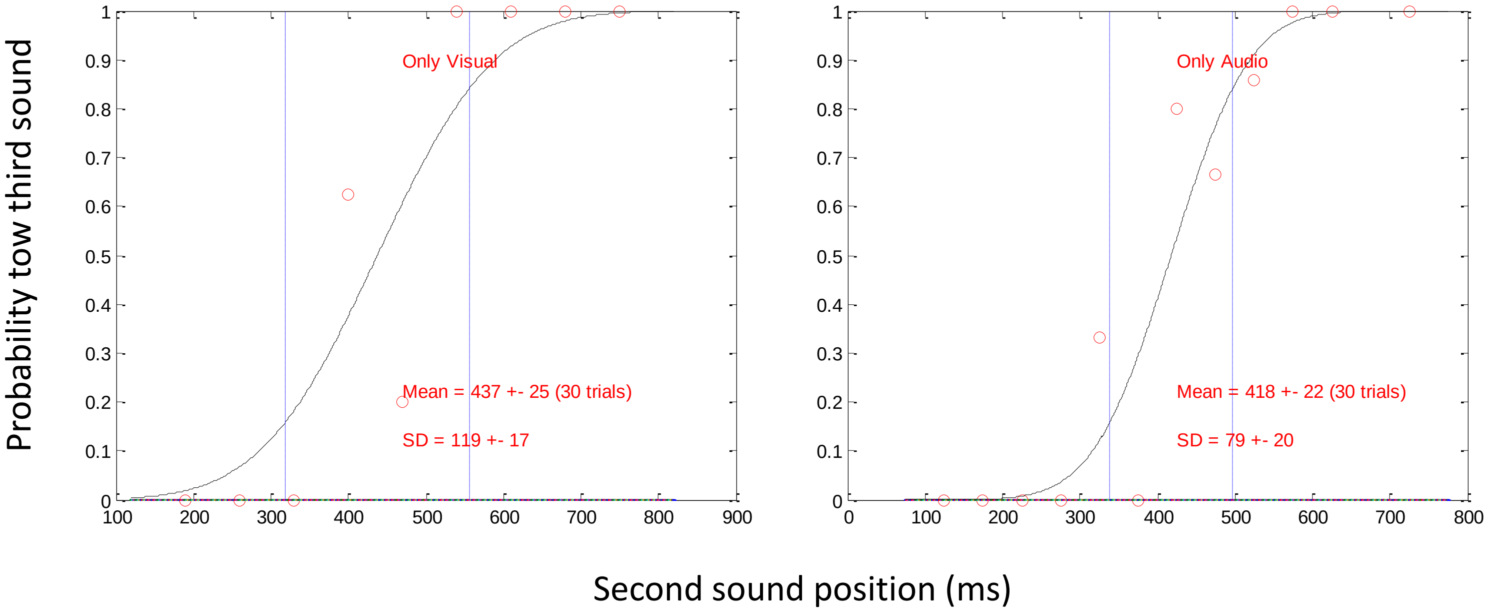

Bimodal

V=50,A=-50

V=0,A=-0

V=-50,A=50

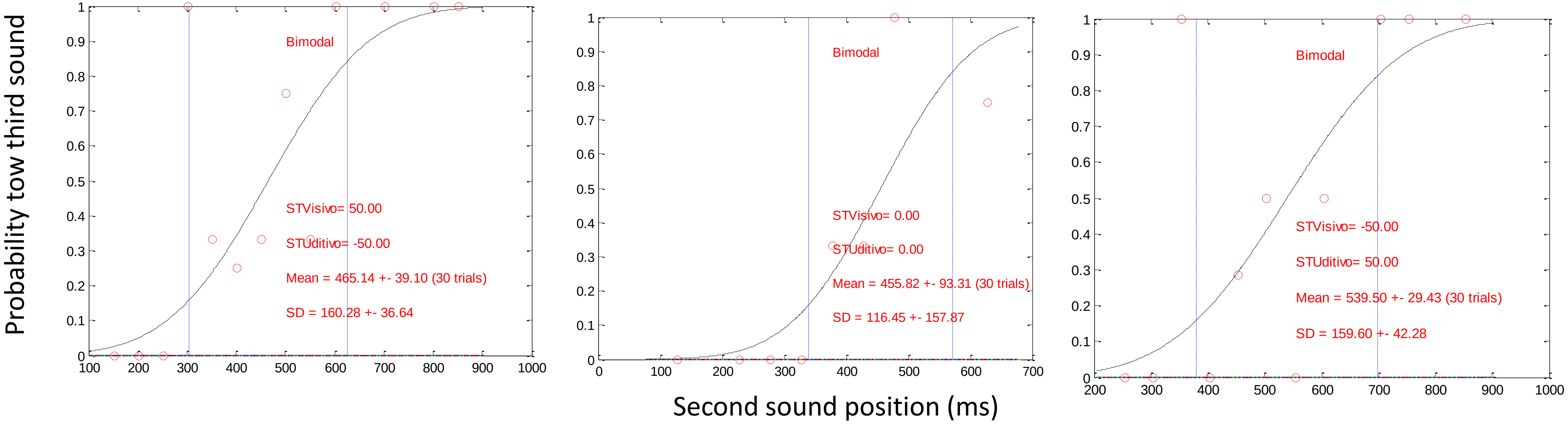

CEx5

Predicted				
	PConf	NoConf	NConf	Beta Bim
	88	90	93	59

OV

OA

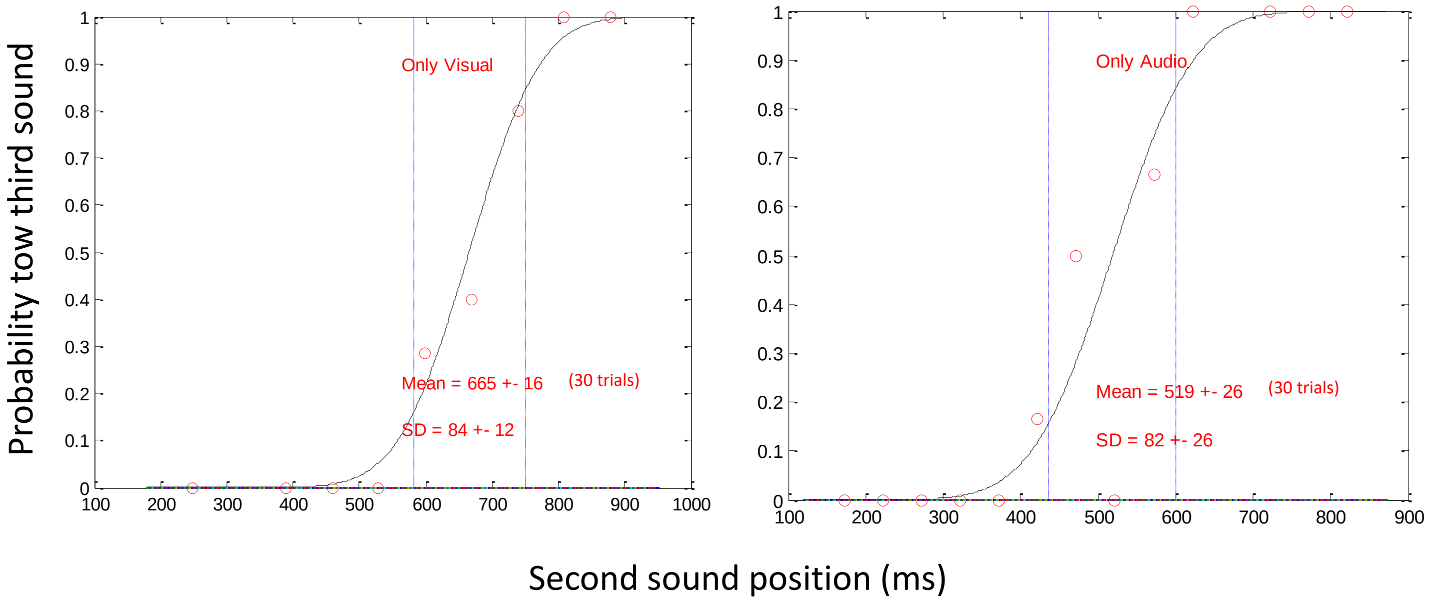

Bimodal

V=50,A=-50

V=0,A=-0

V=-50,A=50

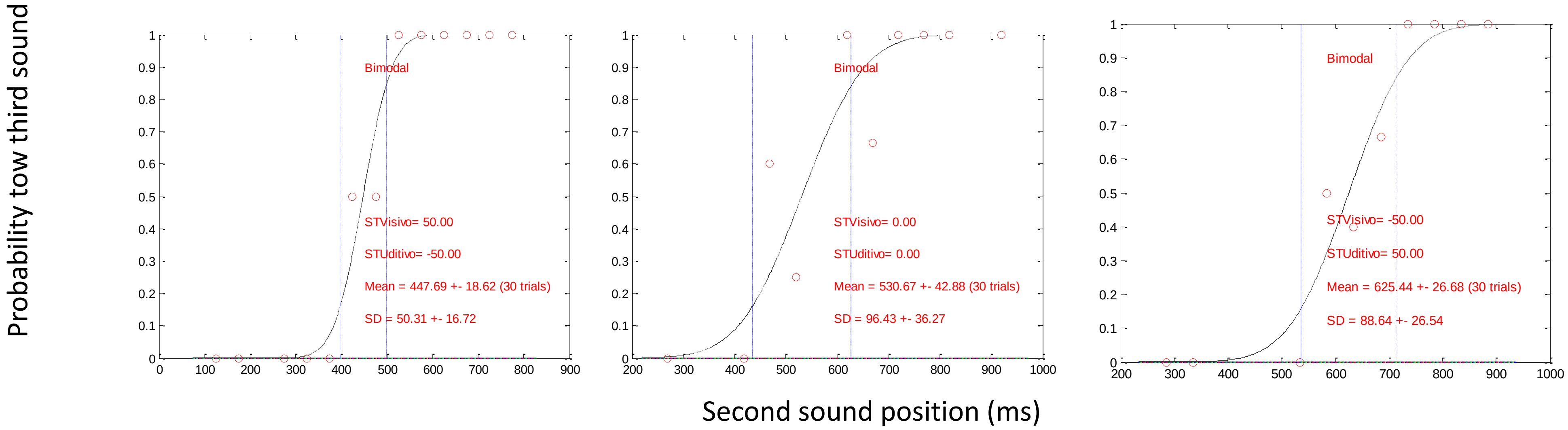

Dyslexic

DYEx1

Predicted				
	**PConf**	**NoConf**	**NConf**	**Beta Bim**
	145	132	119	161

OV

OA

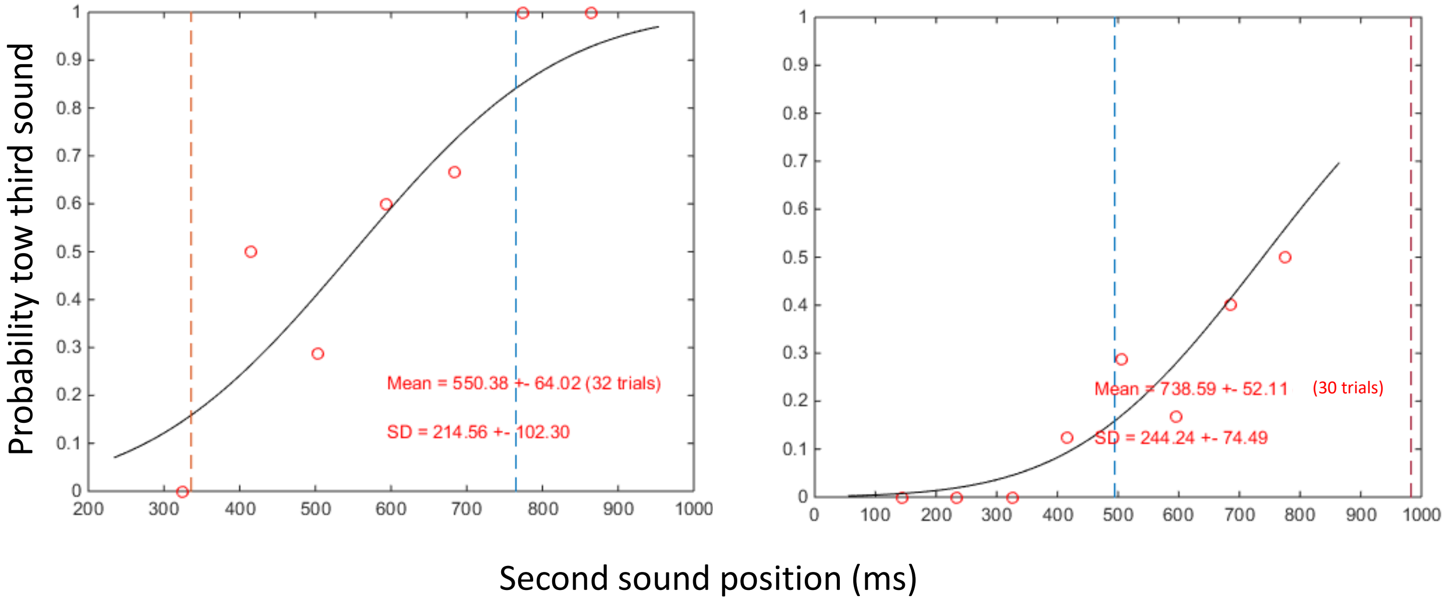

Bimodal

PConf

NoConf

NConf

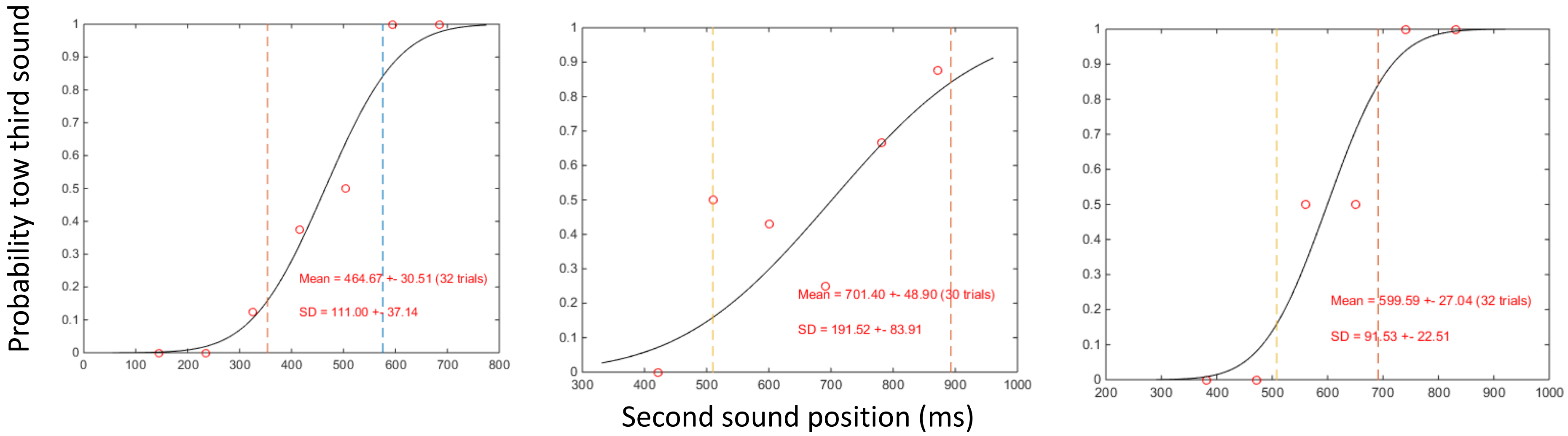

DYEx2

Predicted				
	PConf	NoConf	NConf	Beta Bim
	-1	41	84	205

OV

OA

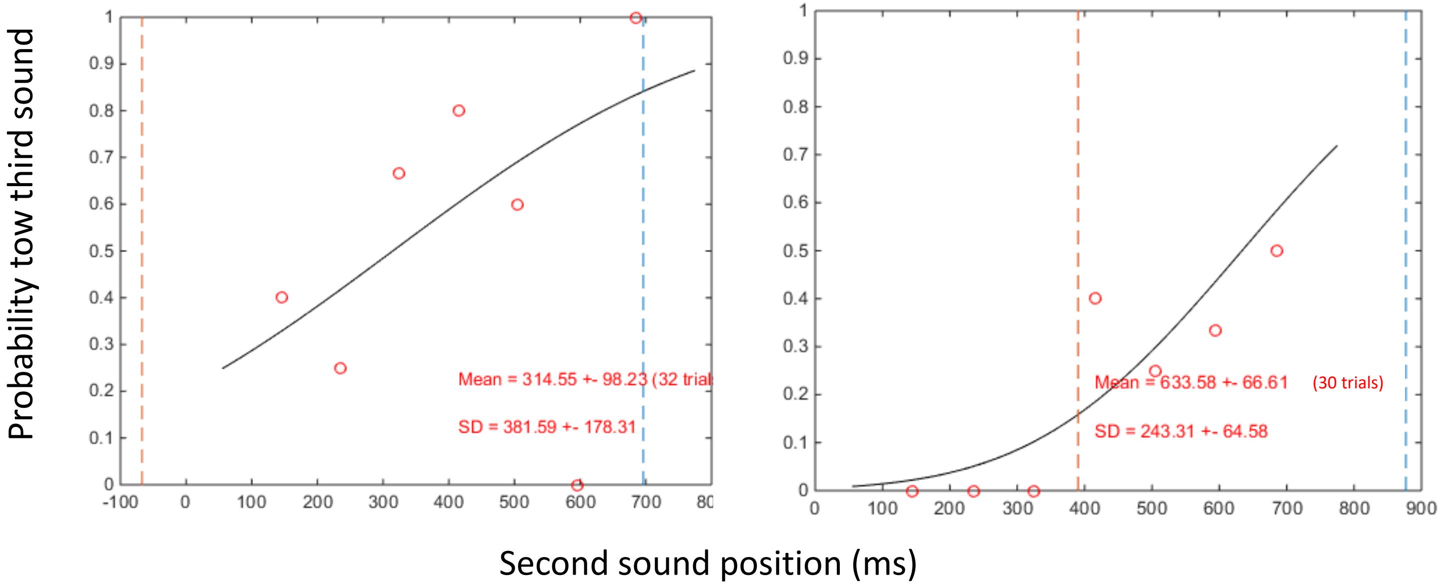

Bimodal

PConf

NoConf

NConf

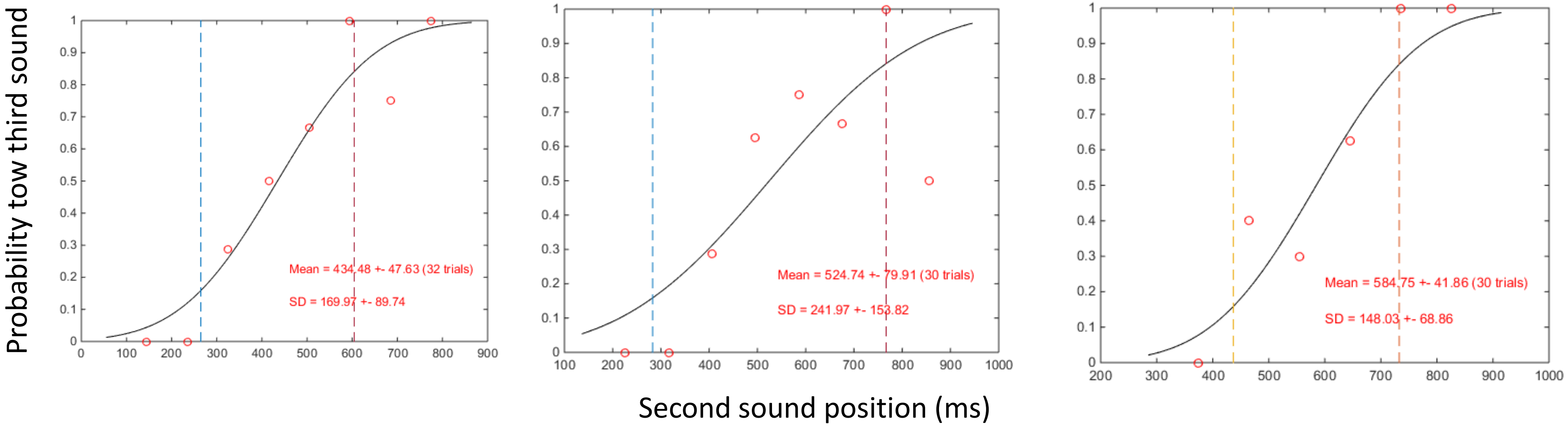

DYEx3

Predicted				
	**PConf**	**NoConf**	**NConf**	**Beta Bim**
	64	116	167	192

OV

OA

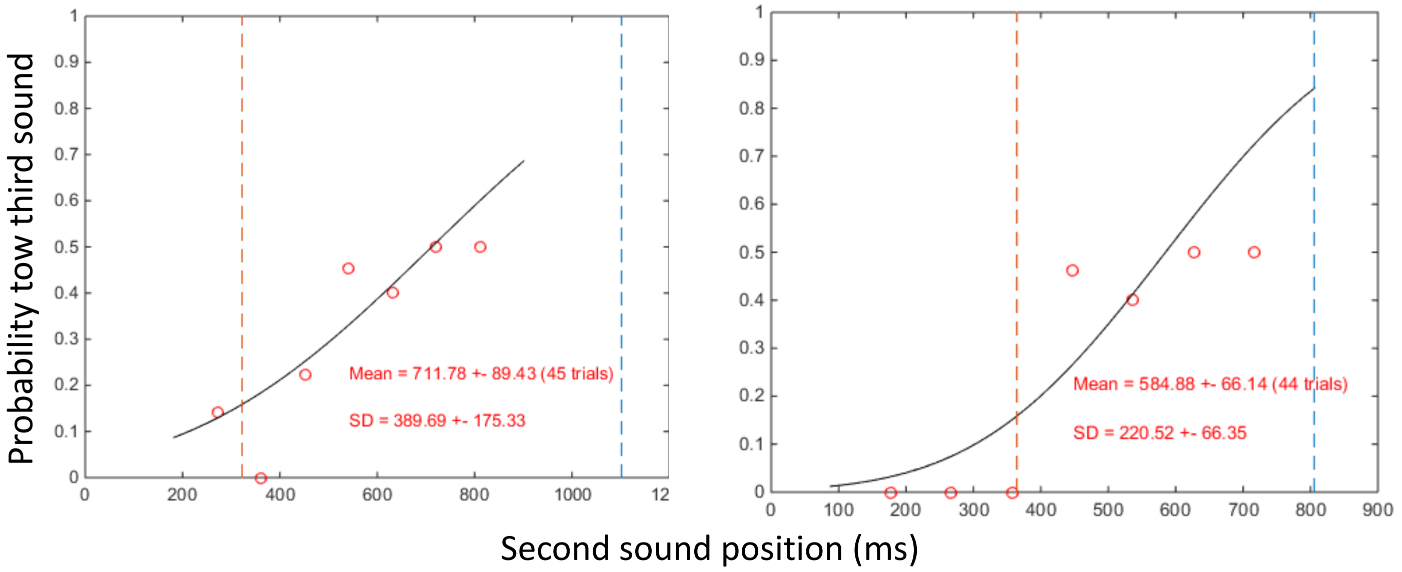

Bimodal

PConf

NoConf

NConf

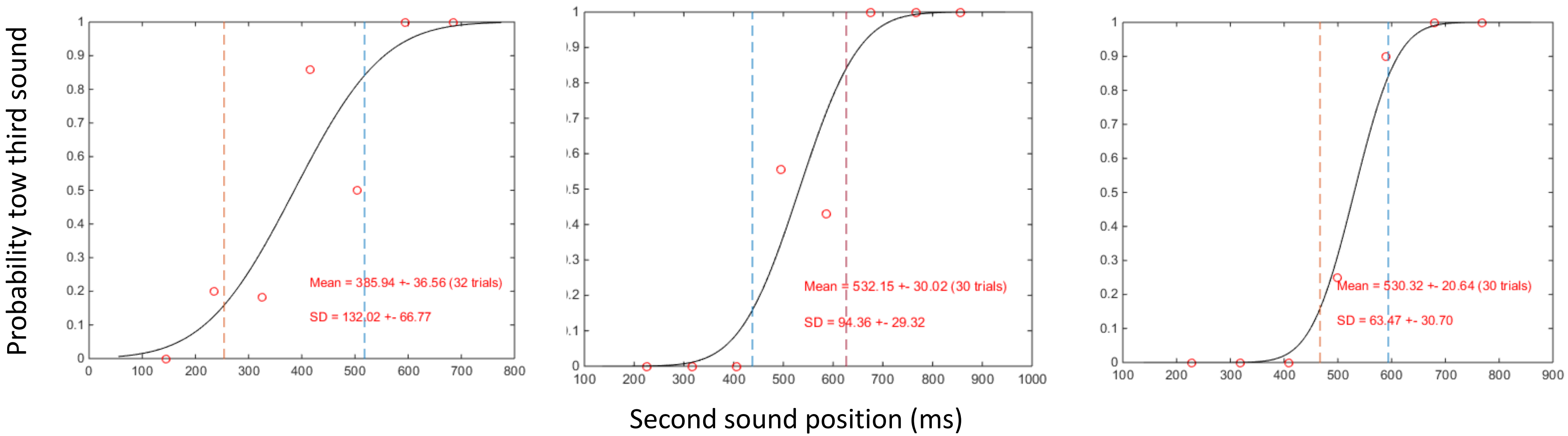

DYEx4

Predicted				
	PConf	NoConf	NConf	Beta Bim
	216	215	214	205

OV

OA

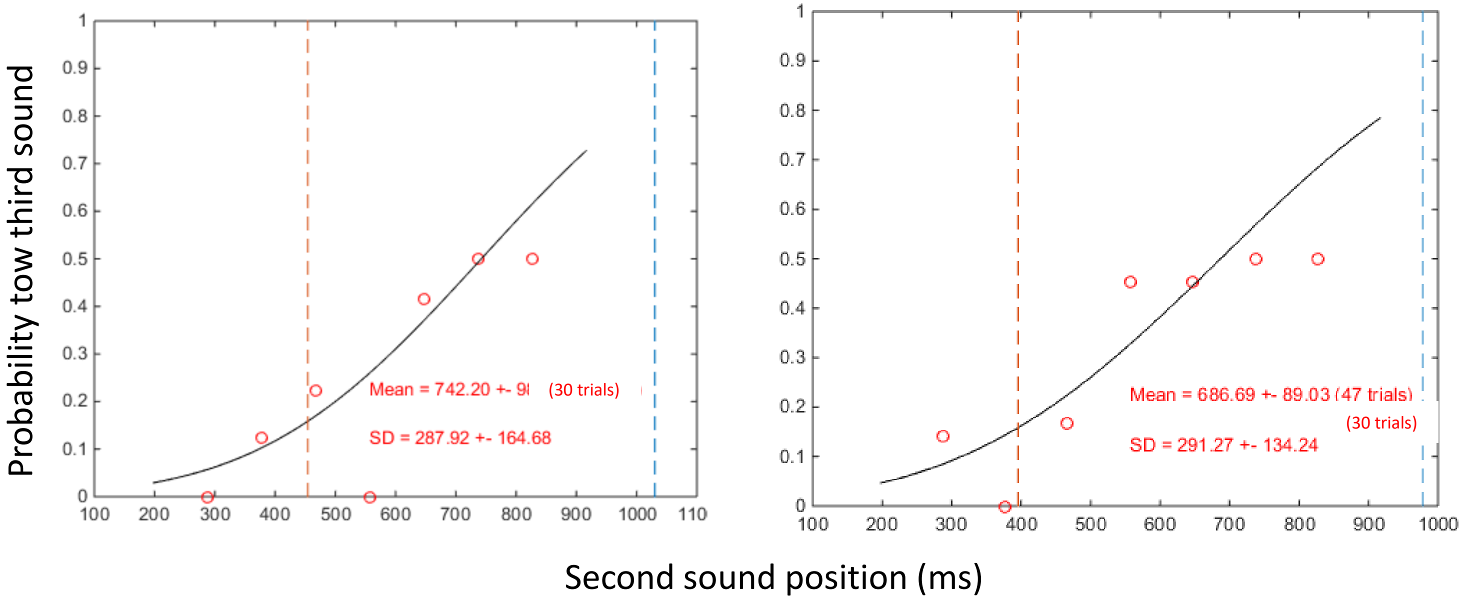

Bimodal

PConf

NoConf

NConf

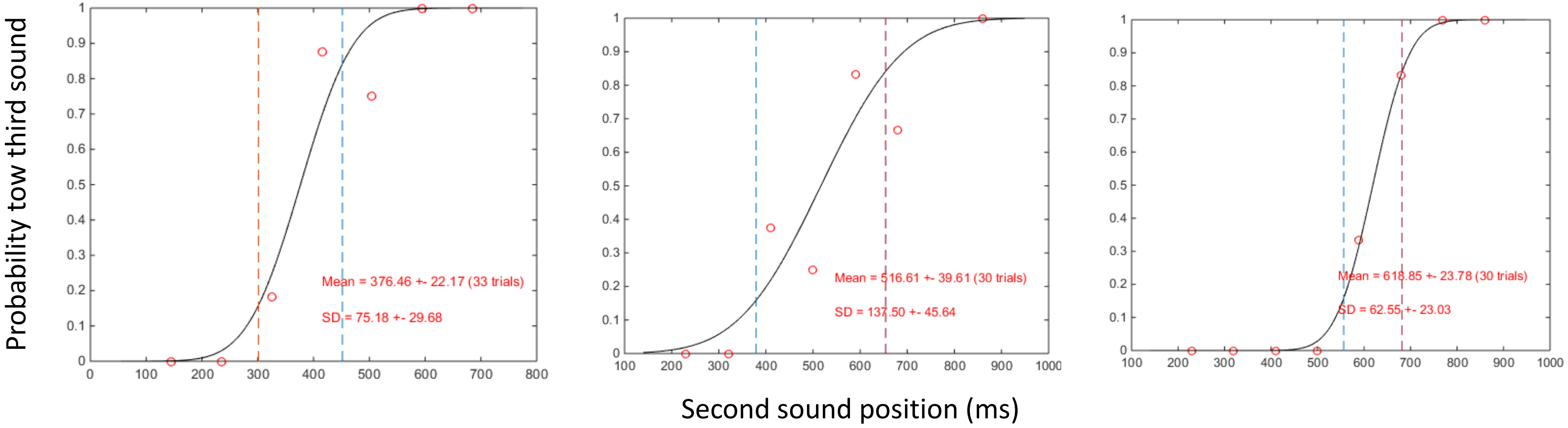

DYEx5

Predicted				
	PConf	NoConf	NConf	Beta Bim
	175	202	228	229

OV

OA

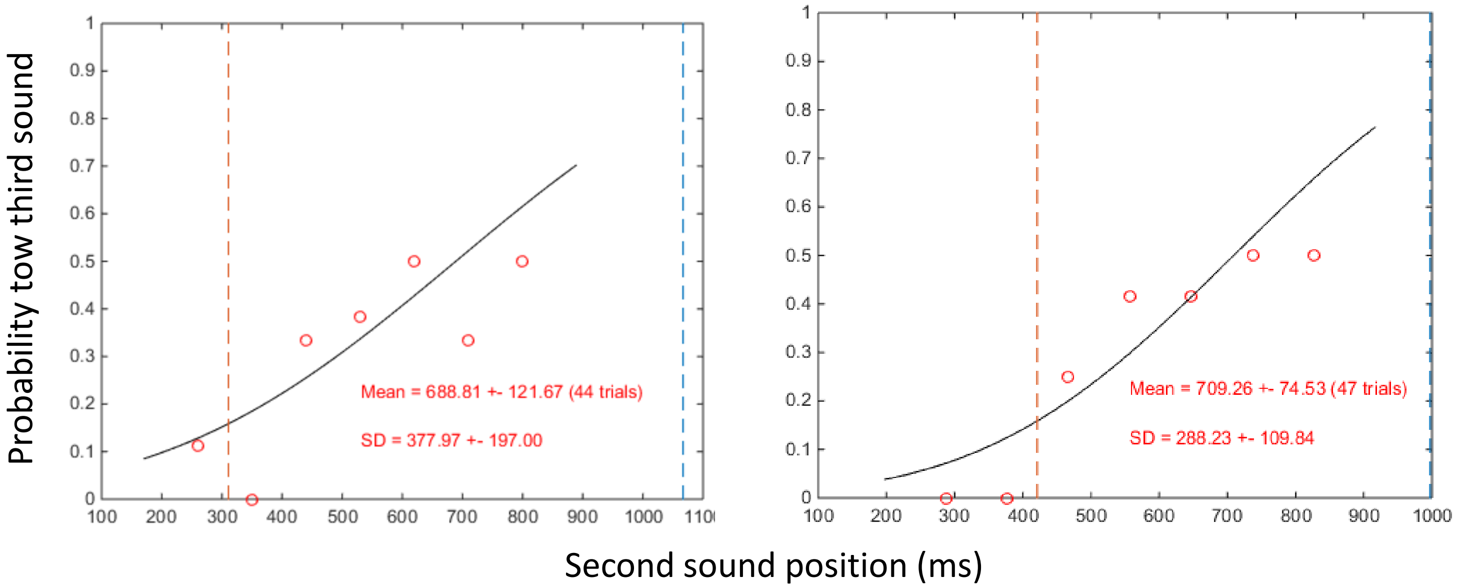

Bimodal

PConf

NoConf

NConf

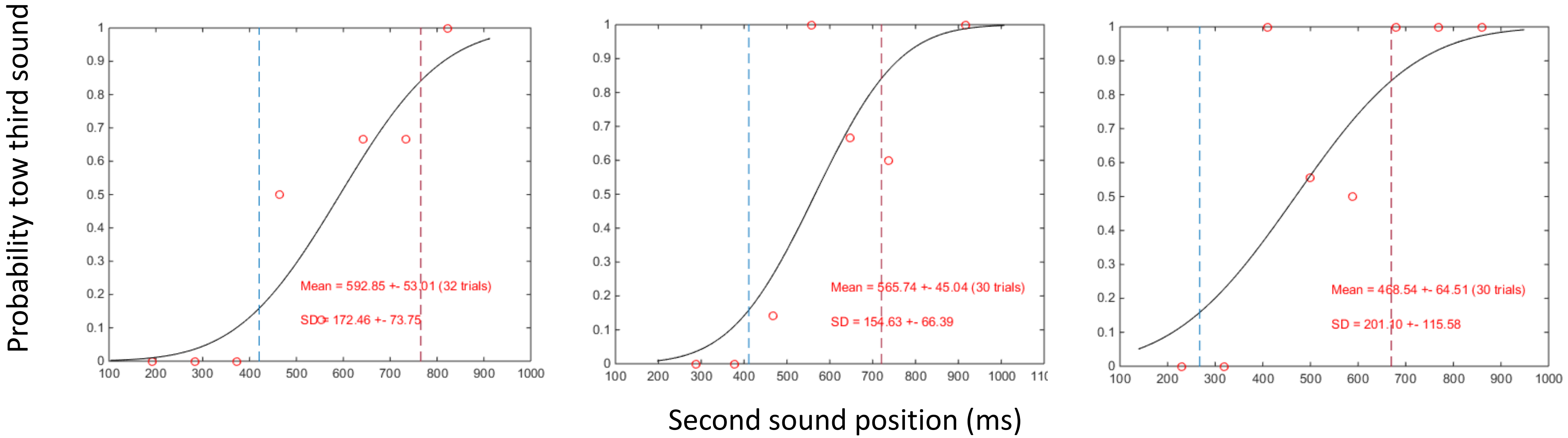

Supplement: Supplementary file 1 — Supplementary Material [file DESC-23-e12977-s001.pdf]
